# The complement system in human pregnancy and preeclampsia

**DOI:** 10.3389/fimmu.2025.1617140

**Published:** 2025-08-19

**Authors:** Vijay Kumar, John H. Stewart

**Affiliations:** Department of Surgery, Laboratory of Tumor Immunology and Immunotherapy, Medical Education Building-C, Morehouse School of Medicine, Atlanta, GA, United States

**Keywords:** human pregnancy, preeclampsia, CS, placenta, immunoregulation, immune homeostasis

## Abstract

Human pregnancy is a complex condition that poses significant challenges for women due to the necessity of a uterus for key processes such as fertilization, embryo implantation, fetal development, and childbirth. These processes are governed by immunological factors and accompanied by various physiological changes. For a successful pregnancy, maternal immune reprogramming is crucial because the developing embryo is considered a semi-allograft. Any immunological alteration during pregnancy induces recurrent pregnancy loss and other fetal–maternal health issues, including preeclampsia. However, despite advances in reproductive immunology, the exact immunopathogenesis of preeclampsia remains unclear. The complement system (CS) is an evolutionarily ancient and critical innate immune component that plays a significant role in maintaining immune homeostasis. The current article discusses the critical role of the CS in human pregnancy and how its dysregulation predisposes pregnant women to preeclampsia. The article introduces the concept of the Th1 to Th2 immunological shift as a prerequisite for a successful pregnancy and the evolution of decidualization via transposable elements, which recruit genes responsible for the process in the endometrium. The immune system plays a critical role in decidualization. The second section discusses the CS signaling pathway, its negative regulators, and the roles of the C3a/C3aR and C5a/C5aR1/C5aR2 or C5L2 axis in immune homeostasis. The third section elaborates on the role of the CS in the establishment of human pregnancy, such as fertilization, implantation, and fetal development. The fourth section describes maternal CS signaling alteration during successful human pregnancy. The fifth section describes the role of CS signaling in preeclampsia, including its systemic and local (placental) alterations and the responsible mechanisms. The article closes with future perspectives and a summary that describes important complement-based approaches for diagnosing and treating preeclampsia.

## 1 Introduction

**Figure 1:**
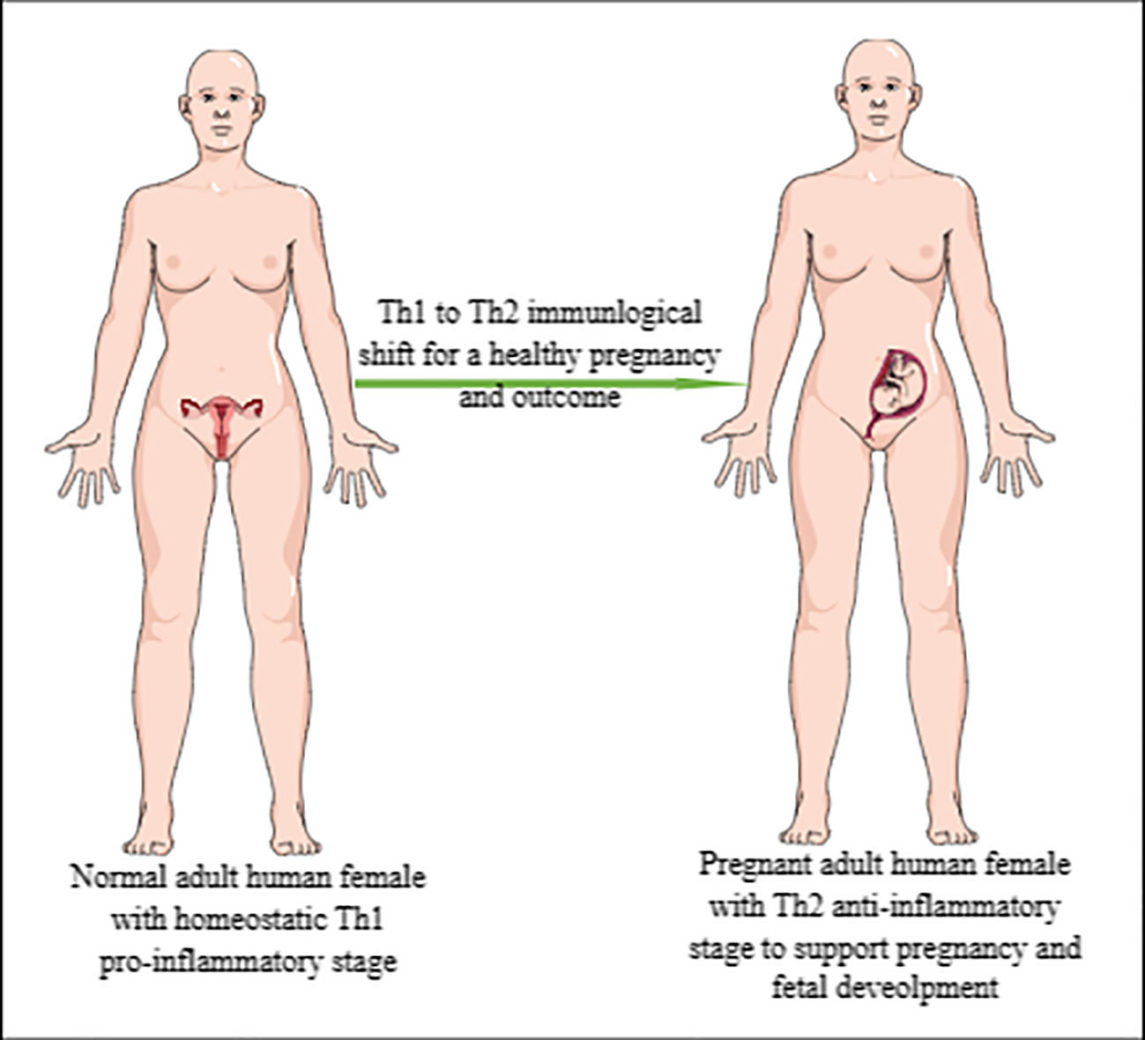
Representation of Th1 to Th2 immunological shift during pregnancy. Normal/healthy non-pregnant adult woman exhibiting pro-inflammatory Th1 immune response to maintain immune homeostasis and fight against invading pathogens and other foreign particles. However, during pregnancy, this pro-inflammatory Th1 immune response shifts to anti-inflammatory Th2 immune response to support pregnancy or developing embryo/fetus, which is an allograft for a pregnant woman.

Human pregnancy occurs in the very specialized organ, the uterus, which protects the developing embryo and fetus through its mucosal lining or decidua, making human pregnancy a unique immune challenge that further develops trained immunity with subsequent pregnancies (1–4). The maternal–fetal interaction during human pregnancy is an example of fetal allograft acceptance by the pregnant female as indicated by the shift from a pro-inflammatory Th1 immune response to an anti-inflammatory Th2 immune response (**Figure 1**) (5, 6). Furthermore, the maternal innate immune system plays a critical role in the successful outcome of human pregnancy. For example, uterine natural killer (uNK) cells are critical for the early embryonic establishment and spiral artery formation (1, 7). Along with the local uterine immune microenvironment, systemic factors, such as hormonal status and cytokine (pro- and anti-inflammatory) levels governing the systemic and local immunological status, determine pregnancy success (1). The details of fetal–maternal immune interactions during human pregnancy have been discussed elsewhere (4, 8–10).

The complement system (CS) is a component of the innate immune system. It is composed of more than 50 humoral components (fluid-phase proteins present in the blood, saliva, lymph, and interstitial fluids), which recognize pathogens and interact with antibodies (Abs)/immunoglobulins (IgG and IgM) and their cognate receptors expressed on different immune cells to maintain immune homeostasis (11, 12). Evolutionarily, the CS is one of the most ancient and primitive components of innate immunity (12, 13). For example, the complement component C3 and factor B genes comprising the central components of the CS originated at least 1,000 million (one billion) years ago (MYA) (13). Furthermore, developmental evolution studies focusing on the origin of pregnancy indicate that the recruitment of genes ancestrally expressed in other organ and tissue systems into endometrial expression transmitted new functions to the uterine endometrium, such as immune regulation and fetal–maternal signaling for a healthy pregnancy (14). The transposable elements (TEs) evolved/amplified prior to the divergence of eutherian mammals were critical for recruiting these genes to the endometrium to induce the development of decidualization, as indicated by the deposition of binding sites for master transcriptional regulators of endometrial stromal cell type identity and progesterone responsiveness to numerous genes across the genome (14). For example, the progesterone receptor (PGR) is the principal transcriptional effector of progesterone signaling and decidualization (14). Thus, decidualization in mammalian pregnancy has also evolved from acquiring genes from other organs required to maintain immune balance for normal functioning. The CS is one of the most ancient components of the innate immune system; therefore, it is critical to understand its role in human pregnancy and preeclampsia.

## 2 CS as a critical component of the immune system

The CS is composed of circulating or humoral components and its receptors called complement receptors (CRs), such as C1q, which is a pattern recognition receptor (PRR) of the complement component C1 (C1 is composed of C1r, C1q, and C1s) and mediates the complement recognition of surface-bound immunoglobulin (Ig) G and IgM, CR1 (CD35), CR2 (CD21), CR3 (CD11b/CD18 or Mac1), CR4 (CD11c/CD18), CRIg (VSIG4, expressed on Kupffer cells and several other tissue-resident macrophages), C3aR, C5aR1 (CD88), and C5aR2 or C5L2 (12, 15, 16). The liver is a major producer of circulating CS components (17, 18). However, epithelial, endothelial, and immune cells, such as neutrophils, monocytes and macrophages, dendritic cells (DCs), mast cells, B cells, and T cells, also produce different CS components or proteins (19). CS activation is a rapid innate immune response against invading pathogens, including microbe/pathogen-associated molecular patterns (MAMPs/PAMPs) and death/damage-associated molecular patterns (DAMPs), aimed at containing the infection and inflammation.

**Figure 2:**
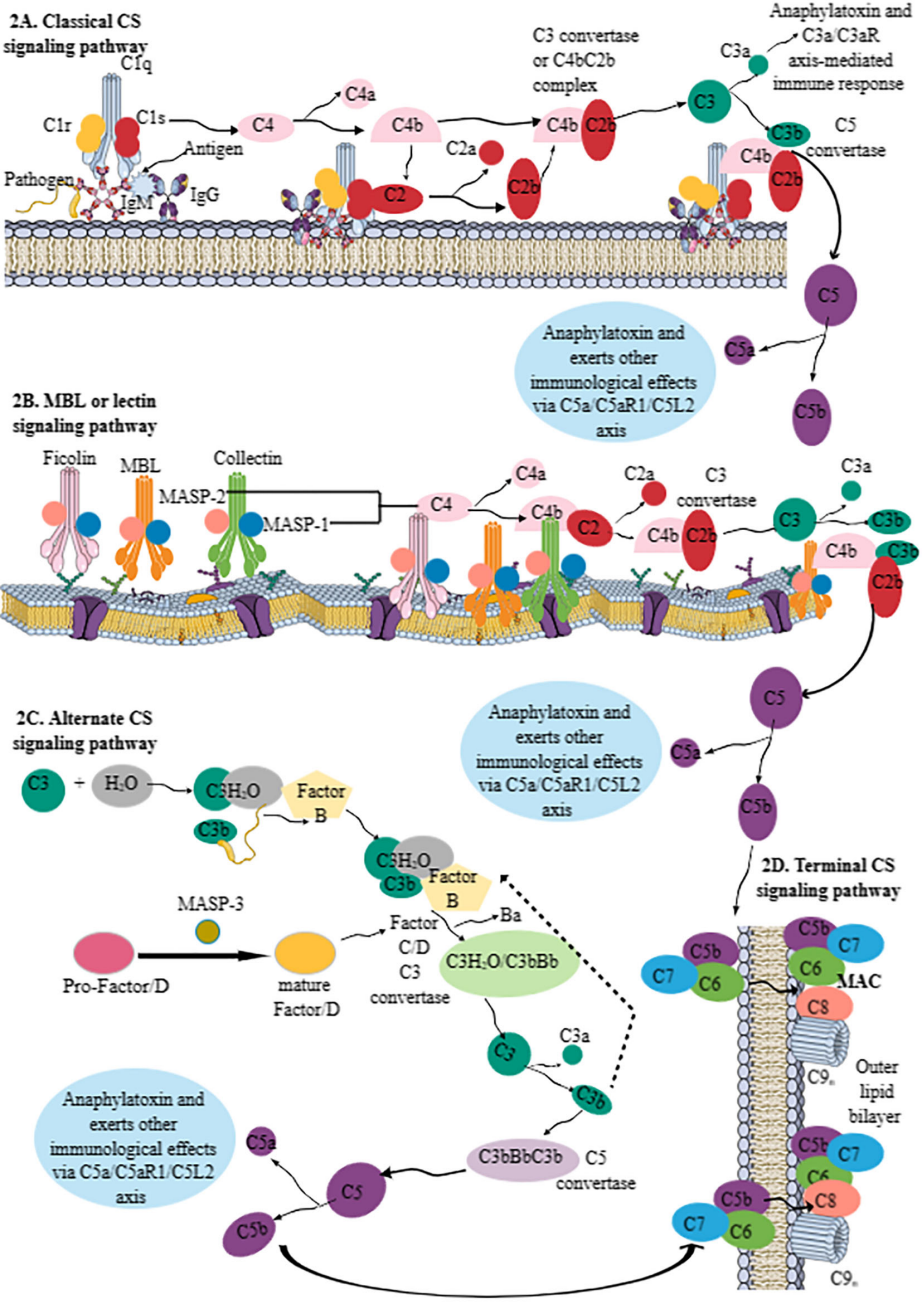
Schematic representation of the CS signaling pathways. **(A)** Classical CS signaling pathway. The C1 component [comprising C1q (serves as a PRR) and 2C1r and 2C1s components, which are serine proteases] initiates the classical CS signaling pathway by recognizing IgG and/or IgM bound to the pathogen, cell surfaces, or other immune complexes. The C1q binding to the pathogen/antigen/IC-IgM/IgG complex activates two serine proteases (C1r and C1s). The activated C1s recognizes C4 and generates C4a and C4b components. C4b recruits C2 and C1s to generate C2a and C2b. The C4bC2b complex serves as *C3 convertase* of the classical CS signaling pathway. *C3 convertase* cleaves C3 into C3a (an anaphylatoxin; alters immune response via C3a/C3aR axis on different immune cells) and C3b (opsonin). The remaining C3b attached to C4bC2b forms C4bC2bC3b complex called *C5 convertase*, which cleaves C5 into C5a (an anaphylatoxin; alters immune response via C3a/C3aR axis on different immune cells) and C5b, which forms MAC by activating the terminal pathway. **(B)** The lectin or MBL pathway. The MBL pathway does not require C1 but instead depends on ficolin-, MBL-, and collectin-mediated pathogen recognition. MASP-1 and MASP-2 of these molecules upon pathogen recognition become active. For example, MASP-1 activation stimulates MASP-2 enzymatic activity for C4 and C2 molecules to generate C4bC2b or lectin pathway *C3 convertase* to generate C3a and C3b. This pathway also generates C5a and C5b, like classical CS signaling pathway, to generate MAC. **(C)** The alternative CS signaling pathway. The alternative CS signaling pathway involves hydroxylation of C3 to form C3(H_2_O) complex, which recognizes circulating pathogens. The bound C3b on the pathogen surface is recognized by FB. The MASP-3 of the MBL pathway cleaves pro-factor D to mature factor D that serves as a serine protease to cleave factor B (FB) and generate the C3 convertases C3(H_2_O)Bb and C3bBb. Thus, the C3(H_2_O)/C3bFB complex generates C3(H_2_O)/C3bBb as a *C3 convertase* of the alternative CS signaling pathway that cleaves C3 into C3a and C3b. The fast production of C3bBbC3b (C4b2b3b) serves as a *C5 convertase* of the alternative CS signaling pathway to generate C5a and C5b. **(D)** The terminal pathway of the CS. C5b, generated due to the activation of all three CS pathways, forms a complex with C6, C7, C8, and C9 components called MAC. MAC kills invading pathogens by forming pores on their cell membranes. Kindly see the text for details. CS, complement system; PRR, pattern recognition receptor; MAC, membrane attack complex; MBL, mannan-binding lectin; FB, factor B.

The CS activation further activates innate immune cells by promoting phagocytosis by producing opsonins, which induce opsonization, and the stimulation of different CRs (C3aR, C5aR1, and C5aR2) expressed on innate and adaptive immune cells, further activating both (innate and adaptive) arms of the immune system to maintain immune homeostasis. The CS activation pathway diverges mainly into three pathways: 1) classical CS activation, 2) lectin or mannan-binding lectin (MBL) pathway, and 3) alternative CS activation (**Figure 2**). It is critical to note that the alternative CS signaling pathway is evolutionarily older and that the classical CS signaling pathway evolved from it (20, 21). Complement component C3 activation is common to all three CS pathways, or all these pathways converge at C3 to form the end product, called the membrane attack complex (MAC), which is composed of C5bC6-9 (**Figure 2**). A brief description of all three CS signaling pathways forming the MAC has been discussed below and is shown in **Figure 2**.

Classical *CS* activation starts from the C1 component, which is composed of three components: C1q, C1r, and C1s (**Figure 2A**). C1q serves as a pattern recognition molecule (PRM) or PRR. It recognizes structural changes induced by IgM and/or IgG1, IgG2, and IgG3 Abs binding to pathogens, cell surfaces, or immune complexes (ICs) (**Figure 2A**). This recognition, or the C1q–pathogen/antigen/IC-IgM/IgG complex, activates two serine proteases (C1r and C1s) of C1. The enzymatically activated C1s recognizes complement component C4 and cleaves it into *C4a* (smaller fraction) and *C4b* (larger fraction) (**Figure 2A**). The biological role of *C4a* is not yet clear, whereas C4b within or near the Ig–C1 complex recruits fluid phase C2 and C1s, which process C2 to *C2a* (biological function unknown) and *C2b* (which is an active serine protease) (**Figure 2A**). The *C4bC2b* complex serves as a classical CS pathway *C3 convertase*, a central player for all three CS pathways (**Figure 2A**). *C3 convertase* promotes C3 activation, a central component of CS signaling pathways. C3 cleavage produces C3a (serves as an anaphylatoxin and induces C3a–C3aR interaction-mediated immune response) (**Figure 2A**), and C3b serves as an opsonin to aid in phagocytosis through its exposed thioester group that recognizes amino or hydroxyl groups on the target (15, 22, 23). The remaining C3b within C4bC2b forms a complex called C4bC2bC3b or *C5 convertase* (**Figure 2A**). The *C2b* component of *C5 convertase* cleaves C5 into C5a (serves as anaphylatoxin and inflammogen and induces C5aR1- and C5aR2-mediated immune functions) and C5b (mediates the activation of terminal pathway or MAC formation) (**Figure 2A**).

The *lectin or MBL pathway* is independent of Abs and shares many characteristics of classical and alternative CS signaling pathways (**Figure 2B**). The MBL or lectin pathway does not require C1 for its activation; instead, it depends on the recognition of PAMPs by MBL, three *ficolins* (*Ficolins 1–3*), and two *collectins* (*Collectin-10* and *Collectin-11*), which have serine protease activity (**Figure 2B**). MBL and collectins (Collectin-10 and Collectin-11), due to their carbohydrate recognition domain, are part of the superfamily of fibrinogen-like proteins, whereas ficolins have a fibrinogen-like recognition domain and belong to the superfamily of fibrinogen-like proteins. Thus, MBL, ficolins, and collectins can recognize different carbohydrate entities, such as mannose of bacterial pathogens by MBL, *N*-acetylglucosamine (GlcNAc) of injured and dying cells by MBL and ficolins, and altered l-fucose and D-galactose patterns of cells under severe stress by ficolins and Collectin-10 and Collectin-11. Furthermore, the lectin pathway can also recognize the host DNA exposed on apoptotic cells, and *Collectin-12* may activate the alternative CS signaling pathway in its soluble form in conjunction with properdin. MBL-associated serine protease-1 (MASP-1), MASP-2, and MASP-3 are MBL or lectin pathway serine proteases. MASP-1 and MASP-2 are associated with common collagen regions within MBL, ficolins, and collectins (**Figure 2B**), whereas MASP-3 activity is connected to the alternative CS pathway activation via the proteolytic cleavage of pro-Factor D (FD) to enzymatically active mature FD (**Figure 2C**). FD is a serine protease that is critical for activating the alternative CS signaling pathway by cleaving factor B (FB) and generating the C3 convertases C3(H_2_O)Bb and C3bBb (discussed later in the alternative CS signaling pathway section) (24, 25). Adipocytes are the main producers of circulating FD (24, 25). The MASP-3-mediated cleavage of FD to its mature form prepares it for its initiation and amplification function of the alternative CS signaling pathway (25). The details of FD in the CS signaling pathway and complement-mediated inflammatory diseases have been discussed elsewhere (24, 25).

The MBL or lectin pathway recognizes the target molecule and autoactivates MASP-1, cleaving MASP-2 for its enzymatic activity toward C4 and C2 bound to initiating MBL, ficolins, and collectin–MASP-1/2 complexes. This is followed by classical CS signaling, such as forming a *C4bC2b* complex and a *C3 convertase*, activating C3 and C5 (**Figure 2B**). The C3 and C5 convertases of the lectin pathway are also known as lectin pathway convertases.

The *alternative CS signaling pathway* has been considered a separate CS activation pathway (**Figure 2C**). However, it can activate the CS itself and may account for approximately 80%–90% of the total complement activation, even in conditions triggered by *classical* or *lectin*/*MBL pathways* (26). For example, it idles in the serum constantly at low levels of activation, as 3%–5% of circulating C3 constantly exists in a hydrolyzed [C3(H_2_O)] form, and its exposed thioester can spontaneously interact with complement targets, such as microbes in the peripheral circulation (blood, lymph, and interstitial fluids). Thus, C3(H_2_O) is bound to the circulating pathogen, and C3b is deposited on the target surface during the *classical* or *lectin/MBL* pathway, recognized by the inactive serine protease, FB (**Figure 2C**). The C3(H_2_O)/C3bFB complex interacts with another serine protease, FC, that cleaves FB into Ba and Bb (enzymatically active) units (**Figure 2C**). The smaller Ba subunit detaches itself from the C3(H_2_O)/C3bFB complex, generating C3(H_2_O)/C3bBb, the *C3 convertase* of the *alternative CS signaling pathway* (**Figure 2C**). The Bb component of *the alternative CS signaling pathway*’s *C3 convertase* cleaves C3 into C3a and C3b, as occurs during *classical* or *lectin/MBL* pathways (**Figure 2C**). The rapid production of C3bBbC3b (C4b2b3b) complexes during the *alternative CS signaling pathway* forms its *C5 convertase*, cleaving C5 into C5a and C5b (**Figure 2C**) (27). *Properdin*, a complement protein, along with stabilizing the *C3 convertase* complexes (C3bBbP) of the *alternative* CS signaling pathway, may also serve as an initiator or focus point for the subsequent C3b deposition during different conditions, such as apoptotic immune cell death as seen during acute infections, such as sepsis (27, 28). For example, *properdin* targets specific proteoglycans of apoptotic immune cells, serving as DAMPs and microbial PAMPs, and recruits C3b, which promotes phagocytic clearance of these pathogens and apoptotic cells (27).

The *terminal pathway* of CS signaling involves the convergence of all three CS signaling pathways that form the final and lytic component of the CS called the MAC, which forms pores in the targeted microbial surface (**Figure 2D**) (27). The MAC is composed C5b, C6, C7, C8, and C9 components of the CS (**Figure 2D**) (27, 29, 30). The complement components C6 to C9 are members of the MAC/perforin/cholesterol-dependent cytolysin (MACPF/CDC) protein superfamily. Thus, the MAC is the *CS signaling* end product that kills the target by forming pores. However, continuous CS activation is governed by several circulating or surface-bound regulators to prevent host damage, as described in **Table 1**.

**Table 1:** Circulating and cell surface/membrane-bound complement regulators.

Soluble/circulating complement regulators	Organ-specific expression	Cell-specific expression	Functions	Deficiency or overexpression diseases
*C1 inhibitor (C1-INH) or C1 esterase inhibitor belongs to the serine protease inhibitor (Serpin) superfamily, also known as Serpin family G member 1 (SERPING1)*	Highly expressed in the lungs, liver, female reproductive tract (FRT), and placenta (31, 32).	Platelet alpha granules and parenchymal cells of the liver, including hepatocytes, Kupffer cells (KCs), blood monocytes, microglial cells, skin fibroblasts, and endothelial cells (31, 33–36).	C1-INH binds to the active sites on both C1r and C1s to form a complex C1-INH-C1r-C1s-C1-INH for inhibiting activated C1 (37). It also inhibits C1 autoactivation by removing the intact C1qrs complex (38, 39). C1-INH also inhibits kallikrein, plasmin (fibrinolytic), and contact activation (intrinsic) coagulation cascade (40, 41). C1-INH also regulates alternative CS pathway (42). CI-INH also inhibits endothelial cell and leukocyte interaction by interacting with P- and E-selectin during inflammation (43).	C1 inhibitor deficiency is associated with hereditary angioedema or hereditary angioneurotic edema (HAE), causing severe swelling in the body/specific body parts due to leakage of fluids in the connective tissues from blood vessels as result of mild trauma (44).
*Complement Factor I (CFI) or C3b/C4b inactivator*	Mainly (90%) synthesized in the liver and circulates as a zymogen till the engagement of C3b/cofactor complex; eyes (in aqueous and vitreous fluid) (45).	Hepatocytes, retinal pigment epithelial cells (RPECs), monocytes, endothelial cells, keratinocytes, myoblasts, and hepatoma cells (45).	CFI inhibits activated C3b and C4b in the presence of cofactors, like Factor H, C4b binding protein, complement receptor 1 (CR1 or CD35), and membrane cofactor protein (MCP or CD46) (45, 46). CFI also inhibits the alternative CS signaling pathway at cellular surfaces expressing CD46 and CR1 (47).	Genetic CFI deficiency is associated with recurrent infections with encapsulated bacteria (i.e., *Neisseria meningitidis*, *Haemophilus influenzae*, and *Streptococcus pneumoniae*), renal autoimmune diseases, such as C3 glomerulopathy (C3G), atypical hemolytic uremic syndrome (aHUS), and age-related macular degeneration (AMD) (45, 46).
*Complement Factor H (CFH) or Factor H (FH)*	Mainly synthesized by the liver.	Hepatocytes, monocytes, dendritic cells (DCs), endothelial cells, fibroblasts, RPECs, and keratinocytes (48).	Mainly inhibit alternative CS signaling pathway systemically and cellular level (48–51). FH serves as an alternative CS signaling pathway convertase decay accelerator by binding to the C3b via its N-terminal four domains and FI cofactor to inactivate C3b (52). Thus, FH prevents the formation of C3bBb convertase and accelerates its decay (48). FH also inhibits classical CS signaling pathway activation in response to the fibrin clot (53).	Quantitative FH deficiency is associated with C3G and mutations in the FH complement regulatory N-terminal domains, also associated with C3G, whereas C-terminal mutations are associated with defective surface recognition and aHUS (48). Some FH genetic variants are also associated with AMD (48, 54, 55).
*Factor H-related protein (CFHR) family has five family members (CFHR1–CFHR5). CFHRs are exclusively composed of complement control protein (CCP) domains*, *which are also called Sushi domains or short consensus repeats (CSRs)* (48, 56)	Liver	Hepatocytes, monocytes, dendritic cells (DCs), endothelial cells, fibroblasts, RPECs, and keratinocytes (48).	Their function is less characterized and controversial (57). However, all five CFHRs (CFHR1–CFHR5) bind to C3b and compete with FH in the process. For example, CFHR1, CFHR4, and CFHR5 interact with C3b, enhancing alternative CS signaling pathway activation as CFHR4–C3b complex binds to factor B and properdin, forming an alternative convertase to cleave C3 into C3a and C3b (48, 57, 58).	CFHR variants are associated with increased risk for aHUS (59). Malondialdehyde (MDA)-bound CFHR1 increases pro-inflammatory cytokine release from immune cells, such as macrophages, without involving CS (60). However, CFHR1 deficiency increases TNF-α, IL-1β, and IL-6 release from lipopolysaccharide (LPS) and R484-stimulated monocytes (60).
*C4b-binding protein (C4BPA and C4BPB)*, the *only complement inhibitor with a polymorphic structure* (61)	Mainly by liver and to some extent by the lungs and cells of islets of pancreas (62).	Hepatocytes, granulocytes, and monocytes	CRBP prevents uncontrolled activation of classical and MBL/lectin CS signaling pathways by serving as a cofactor for CFI during C4b inactivation and prevents the classical *C3 convertase* (*C4bC2a*) formation, also promoting the *C3 convertase* decay by preventing *C3 convertase* binding to the nascent C4b (61, 62). C4BP also inhibits *alternative CS* signaling pathway in the fluid phase by serving as a cofactor for CFI without inhibiting alternative *C3 convertase* (63–65). C-reactive protein (CRP)–C4BP complex also inhibits classic CS activation (66). C4BP also promotes apoptotic cell death (ACD) (62). CRBP also serves as an acute-phase reactant, and it increases several-fold during inflammatory conditions (61). C4BP binding to pathogens helps them to escape from complement-dependent clearance. However, C4BP inhibits target cell entry of H1N1 influenza A virus (IAV) and provides protection against infection (67). C4BP also inhibits silica and/or monosodium urate (MSU)-induced NLRP3 inflammasome activation in macrophages (68).	Confirmed cases of C4BP deficiency have not yet been reported in humans. Only one human case has been reported of the primary C4BP deficiency in a patient with disease clinically resembling Behçet’s disease with angioedema (69), but it is not clear whether the complete disease was associated with C4BP deficiency or just the angioedema.
*Properdin is a plasma glycoprotein and the only known positive regulator of the CS*, *especially alternative CS signaling pathway* (70, 71).	Liver immune cells, but not by hepatocytes (72).	Synthesized mainly by monocytes–macrophages, DCs, T cells, and granulocytes, including mast cells (70). Adipocytes and stimulated endothelial cells are secondary properdin producers (72).	Properdin stabilizes alternative CS signaling pathway *C3 convertase* (C3bBb) by promoting C3b–factor B interaction and providing a focal point for the assembly of C3bBb on a target surface and immune complexes (28, 73). It also binds and stabilizes the preformed alternative CS signaling pathway *C3 convertase* (28). Properdin also inhibits Factor H-mediated cleavage of C3b by FI. Additionally, properdin also stabilizes *C5 convertase* without changing its substrate specificity (74).	Humans with genetic deficiency of properdin are more prone to develop lethal pyogenic infections, especially caused by *Neisseria* spp (75–78).. Interestingly, human properdin deficiency is the only X-linked complement deficiency (72, 79).
*Clusterin*, *also called complement lysis inhibitor (CLI)*, *SP-40*, *40, and apolipoprotein J*	Expressed in variety of tissues and organs (80).	Versatile cells (80)	Inhibits MAC formation by binding to C5b-7 complex in fluid phase (80). It also binds C7, C8β, and domain b of C9 to inhibit MAC formation (80, 81). Furthermore, together with vitronectin, clusterin binds to the nascent amphiphilic C5b-9 complex, rendering it water-soluble and inactive (80).	Systemic lupus erythematosus (SLE) patients with high circulating clusterin and vitronectin greatly demonstrate renal involvement (82). Increased clusterin and vitronectin levels have also been reported in lenses of patients with exfoliation glaucoma (83). Thus, clusterin may not be physiological complement regulator, as it has failed to protect against complement attack under normal or pathological conditions (84).
*Vitronectin*, *also called S-protein and epibolin, was identified as a serum* sp*reading factor and is highly present in plasma* (85).	It is also expressed in different human tissues and organs, such as liver, tonsils, duodenum, heart, skeletal muscles, and lung tissues (85).	Hepatocytes	It binds C5b-7 complex to inhibit MAC formation (85). It also inhibits C9 polymerization (86).	No genetic deficiency of vitronectin has been reported in humans (87). Vitronectin levels impact severity of bacterial infections by different mechanisms, including affecting CS (88). In mice, systemic vitronectin deficiency delays dermal wound healing, increases areas of delayed hemorrhage, and attenuates hepatic fibrosis in non-alcoholic steatohepatitis mouse model (89, 90).
Surface-bound complement regulators	Organ-specific expression	Cell-specific expression	Functions	Deficiency or overexpression diseases
*Complement receptor type 1 (CR1 or CD35 or CD3b/CD4b receptor)*	Wide tissue/organ distribution.	Nucleated cells, immune cells, and red blood cells (RBCs) (91, 92).	It serves as a classical and alternative CS signaling pathway convertase decay accelerator and a cofactor for FI by binding to C3b, iC3b, C4b, and C1q. Hence, CD35 helps in clearance of immune complexes, enhances phagocytosis, and regulates C3 cleavage. CR1 serves as a high-avidity receptor for polyvalent binding to clustered ligands on immune complexes (92).	CD35 genetic deficiency is rare in humans and increases their chances of getting bacterial infections and autoimmunity, such as SLE (93).
*CR2 or CD21*	Organs with B cells and thymus and secondary lymphoid organs (SLOs) expressing follicular DC (FDCs), such as lymph nodes, human palatine tonsils, spleen, and mucosa-associated lymphoid tissue (MALT) (94).	CR2 expression is restricted to B cells and FDCs (93), but subsets of peripheral and thymic T cells also express CR2 (95).	It binds iC3b/C3dg/C3d (96), serves as a B-cell receptor (BCR) co-stimulator, and regulates B-cell function. It also serves as an Epstein–Barr virus (EBV) receptor and binds to different forms of DNAs derived from bacteria, viruses, and mammals with moderate affinity to elicit immune response (95, 97). In humans, B cells’ co-engagement of BCR and CR2 inhibits their proliferation and cytokine and antibody production (98).	SLE patients show reduced CR2 expression, and complete CR1 and CR2 deficiency in mice promotes anti-DNA antibody development in mouse models of SLE (95).
*CR3 or CD11b/CD18 or macrophage antigen-1 (Mac1)*	Not applicable	Expressed exclusively on myeloid immune cells (MICs, such as macrophages, neutrophils, and DCs), NK cells, T cells, and B cells (99–103).	CR3 binds C3d and iC3b fragments of the C3 and promotes phagocytosis of complement-opsonized antigens/cells/molecules by macrophages (92, 104, 105). However, CR3 also involves complement-independent Ab-dependent phagocytosis of fungi, such as *Cryptococcus neoformans* (106). Talin is required for CR3-mediated phagocytosis (107). CR3 expressed on NK cells is responsible for complement-mediated NK cell cytotoxicity toward Ab-coated cancer cells (99, 102).	Genetic CR3 deficiency in humans is associated with severe MIC, such as neutrophils and macrophages, and lymphocyte defects and recurrent bacterial infections (92, 108).
*CR4 or CD11c/CD18 or αXβ2 integrin*	Not applicable	Expressed on MICs, platelets, T cells, and NK cells (103).	CR4 binds iC3b and regulates iC3b-mediated phagocytosis. CR4 on human DCs is critical for inducing early antiviral immune response against complement-opsonized HIV-1 (109).	
*Complement receptor immunoglobulin-like (CR1g) or V-set and Ig domain–containing 4 (VSIG4)*	Liver and peritoneal macrophages	Kupffer cells, monocytes and macrophages, and T cells	CR1g negatively regulates complement activation by the alternative CS pathway by blocking the interaction of C3b in its *C3 convertase* with C5 (92, 110). CRIg enhances phagocytosis of complement-opsonized particles by phagocytes or macrophages (110, 111). CRIg also serves as a direct pattern recognition receptor (PRR) to clear circulating Gram-positive bacteria (112). In T cells, CRIg also serves as an immune checkpoint inhibitor (ICI) like programmed cell death-1 (PD-1) or cytotoxic T-lymphocyte-associated antigen-4 (CTLA-4) and inhibits T-cell proliferation and IL-2 secretion (113).	The genetic deficiency of CR4 is associated with recurrent bacterial infections, and patients die during childhood unless they receive allogenic bone marrow transplantation (92). It is common in patients with primary immunodeficiency, with leukocyte adhesion deficiency type 1 (LAD1), lacking four integrins, including CR3 and CR4 (114).
*CD46 or membrane cofactor protein (MCP)*	Expressed ubiquitously	Expressed on every cell type except RBCs (115).	CD46 is a transmembrane complement regulatory protein, which binds C3b and C4b, and acts as a cofactor for serine protease factor I (FI)-mediated breakdown of C3b and C4b to prevent their immunological function (115). It is expressed by most cells in four isoforms generated due to alternative splicing, and its gene is located in the regulators of complement activation (RCA) gene cluster of the long arm of chromosome 1 (115, 116).	Currently, more than 60 disease-associated CD46 mutations are known; most are linked to aHUS and other diseases, such as SLE, glomerulonephritis, and pregnancy-associated disorders (115–118).
*CD55 or decay accelerator factor (DAF)*, *a glycosylphosphatidylinositol (GPI)-linked membrane protein*	Present ubiquitously, including blood and stroma. It is also present in tears, saliva, urine, and synovial fluids (119, 120).	Epithelial cells, endothelial cells, and RBCs (120).	Recognizes complement component C3b and C4b and inhibits CS signaling pathways (classical, lectin, and alternative). CD55 also serves as receptor for Coxsackieviruses, other enteroviruses, and malaria parasite. CD55 binding to the HIV-1 and hepatitis-C virus (HCV) surface as result of their replication in the infected cells protects them from CS-mediated lysis (121, 122). CD55-CD97 (expressed on macrophages and granulocytes) interaction promotes T- and B-cell proliferation and secretion of IL-10 and GM-CSF (123). CD55 also inhibits NK cell cytotoxic function (124).	Genetic CD55 deficiency is associated with paroxysmal nocturnal hemoglobinuria (PNH) (125). Additionally, genetic CD55 deficiency is also associated with hyperactivation of complement and angiopathic thrombosis, and PLE (the CHAPLE syndrome) has been identified as a monogenic form of primary intestinal lymphangiectasia or Waldmann’s disease (WD) (126). The acquired CD59 deficiency is also associated with autoimmune hemolytic anemia (AHA), autoimmune thrombocytopenia (ATP), and systemic lupus lymphopenia (SLL) (127)
*CD59 or membrane inhibitor of reactive lysis (MIRL) or protectin*, *single chain*, *GPI-linked membrane protein*	Ubiquitous expression, also present in tears and saliva (119, 128).	Widely expressed in cells from all tissues (128). Also expressed on RBCs (128).	CD59 binds C8 and C9 and prevents MAC formation (129). Its binding to HIV-1 also prevents the complement-dependent virus lysis (130).	Genetic CD59 deficiency is associated with PNH (125). The acquired CD59 deficiency is also associated with AHA, ATP, and SLL (127).
*C1qRp or CD93*	Widely expressed.	Prominently expressed by endothelial cells and the MIC, such as monocytes, macrophages, and neutrophils. However, C1qRp expression on monocyte/macrophage is target organ-dependent (131, 132). Platelets and B cells also express C1qRp. C1qRp is critical for Ab secretion and plasma cell maintenance in the bone marrow (133). Naïve T lymphocytes of human neonatal cord blood also express C1qRp (134). CD93 maintains endothelial barrier function or vascular integrity and limits metastasis (135). C1q, MBL, and surfactant protein A (SPA) are C1qRp ligands (132).	C1qRp is not a critical receptor for complement component C1q (131). It is important for phagocytosis of apoptotic cells but not critical for C1q-dependent phagocytosis (136). However, C1qRP enhances phagocytosis of molecules coated with C1q, SPA, and MBL by macrophages (137).	CD93 single-nucleotide polymorphisms (SNPs) and mutations have been found to be associated with autoimmune diseases (AIDs), such as psoriasis and AMD (138).
*C1qRO2−* (139)	Not clear.	Polymorphonuclear leukocytes (PMNLs) or neutrophils and monocytes (132). The C1qRO2− expression on smooth muscle cells is questionable (132, 140).	Triggers reactive oxygen species (ROS), such as superoxide generation in response to C1q binding (132). Inhibits C1 activation (140).	Unknown.
*cC1qR or cell-surface calreticulin (CALR) or collectin receptor* (141)	Wide tissue distribution (141).	Expressed in many cell types intracellularly (endoplasmic reticulum and nucleus) and extracellularly. Human B cells, monocytes, neutrophils, tonsil lymphocytes, vascular endothelial cells, fibroblasts, mesenchymal stem cells (MSCs), and amniotic and pulmonary epithelial cells (141, 142). Not expressed in RBCs.	Binds to C1q-collagen region, and collectins and induces phagocytosis and IL-12 production in antigen-presenting cells (APCs). It also inactivates *C3* and *C5 convertases* (140).	AutoAbs in patients with SLE and Sjögren syndrome (140).
*Megalin*, *an α_2_-macroglobulin receptor family or low-density lipoprotein (LDL) gene family*, *also called epithelial glycoprotein gp330* (141)	Expression is restrictive to absorptive epithelia, such as renal proximal tubules, lung (type II pneumocytes), yolk sac, thyroid, choroid plexus, mammary gland, retina, and inner ear (141).	Epithelial cells.	Binds to C1q and other proteins, such as clusterin, plasminogen activators (free or in complex with their type-1 inhibitor), lipoprotein lipase, apolipoprotein E, and the receptor-associated protein (RAP). It is concentrated in coated pits and performs endocytosis of many proteins (141). Thus, it can endocytose immune complexes by binding to C1q and also clear extra C1q produced during inflammatory conditions seen in organs/tissues expressing megalin to prevent excessive inflammation (141). N-Glycosylation of megalin can modify its ligand-binding activity (143).	Unknown.
*gC1qr* (139)	Widely expressed.	Expressed intracellularly in mitochondria in all mammalian cells except RBCs (140).	Binds to the globular heads of C1q under physiological conditions, inhibits C1 activation but does not interact with collectin (140, 141), serves as a phagocytosis receptor, and modulates mitochondrial function.	Unknown.
*CD305 or leukocyte-associated Ig receptor-1 (LAIR-1)* (16)	Lungs, central nervous system, or CNS.	Immune cells, such as macrophages and DCs, cytotoxic T cells, and type 1 innate lymphoid cells (ILC2s) (144).	Binds C1q and collagens and inhibits DC differentiation and activation and macrophage activation (16, 145–148). Binding of C1q to the LAIR-1 of ILC2s inhibits their pro-inflammatory activity in airway hyperactivity (AHR) seen during type 2 asthma (149, 150).	LAIR deficiency increases neutrophil infiltration in the lungs and lung resistance and permeability (145, 151).
*Specific intercellular adhesion molecule (ICAM)-3-grabbing nonintegrin-related 1 (SIGN-R1 or CD209b*, *a 325-amino acid C-type lectin)*, *which recognizes bacterial dextrans and capsular pneumococcal polysaccharide (CPS) of S. pneumoniae* (152, 153).	Spleen, lymph nodes, peritoneal macrophages, immature DCs, and microglia (154–156).	Splenic marginal zone macrophages (MZMs) and lymph node macrophages (LNMs) (153, 157, 158).	Binds C1q and activates unusual C3 activation-dependent classical CS pathway (independent of Ag–Ab complex-mediated C1q activation) upon recognizing circulating endogenous and microbial (*S. pneumoniae*) polysaccharides (157, 159, 160). SIGN-R1 also cooperates with macrophage CR3 in the phagocytic uptake of oligomannose-coated liposomes (OMLs) (154). MZMs’ SIGN-R1 also mediates complement C1q-dependent clearance of circulating apoptotic cells in the spleen marginal zone (161, 162). Microglia SIGN-R1 also recognize *S. pneumoniae* capsular polysaccharide and activate C1q-dependent classical CS pathway to protect against pneumococcal meningitis (156). SIGN-R1^+^ MZMs are also critical for the maturation of germinal center B cells in the spleen (163).	SIGN-R1 deficiency in mice is associated with defective C3 catabolism and increased susceptibility to bacterial infections (157, 164).

### 2.1 CS in immunoregulation and immune homeostasis

The CS was first described as a critical antimicrobial defense component of innate immunity in circulation between 1888 and 1894 (165, 166). As discussed earlier, the activation of three components of the CS in response to different stimuli, such as Ag–Ab complexes, pathogens (PAMPs/MAMPs), and DAMPs, induces the release of different complement proteins, such as C3a, C3b, C5a, and C5b, and the formation of terminal MAC to clear the exo- or endogenous threat. C3a and C5a are critical immunomodulators that, in addition to serving as anaphylatoxins, also function as potent immunomodulatory agents through their cognate receptors (C3aR, C5aR1, and C5aR2 or C5L2), which are expressed on various innate and adaptive immune cells. Therefore, this section discusses the impact of C3a and C5a on immune cells expressing their cognate receptors.

#### 2.1.1 Impact of C3a on various immune cells via direct interaction with C3aR

In addition to the CS signaling events, C3a can also be generated by different systemic proteases, such as thrombin and immune cell-derived cathepsin G and L (a lysosomal protease) (167–169). However, the cathepsin L (CTSL)-mediated cleavage of the C3 into C3a and C3b has been reported intracellularly in T cells, which maintains their survival through intracellular C3a–C3aR interaction-mediated downstream mammalian target of rapamycin complex 1 (mTORC1), Raptor, and p56 signaling, and extracellular C3a–C3aR promotes the generation of pro-inflammatory Th1 cells as indicated by the generation of pro-inflammatory cytokines, such as interferon-γ (IFN-γ) (170, 171). Resting T-cell lysosomes and endosomes contain C3, and its cleavage by the CTSL to generate “tonic” intracellular C3a is critical in maintaining homeostatic T-cell survival. The transfer of this intracellular C3a to the T-cell surface induces the autocrine pro-inflammatory cytokine production of the Th1 phenotype, which has been seen in T cells isolated from patients with autoimmune arthritis (170). The T cells of patients with autoimmune arthritis exhibit overactivated intracellular CS and IFN-γ production that can be blocked by targeting the intracellular CTSL. Furthermore, intracellular C3–C3aR signaling in intestinal Paneth cells [intestinal secretory epithelial cells with innate immune functions, such as the production and secretion of antimicrobial peptides (AMPs) and other immunomodulatory molecules] also regulates their mTORC1 signaling to enhance their intestinal protective function by supporting the expansion of intestinal stem cells (ISCs) in the intestinal crypts during acute inflammatory intestinal injury (172–174).

Interestingly, in contrast to the extracellular/secreted C3, the intracellular C3 generated via alternative translation in the cytosol is non-glycosylated and present in the reduced state. Intracellular non-glycosylated C3 is turned over by the ubiquitin–proteasome system (UPS) (175). Furthermore, C3 can also be retranslocated from the endoplasmic reticulum (ER) to the cytosol and structurally resembles extracellular/secreted C3. Notably, cytosolic C3 also exerts antimicrobial action in epithelial cells by opsonizing invasive pathogens, such as *Staphylococcus aureus*, decreasing the vacuolar escape, and impacting the bacterial survival by presenting the pathogen to phagocytes, such as macrophages (175). Furthermore, the cytosolic C3 in the β cells of the pancreas protects them from IL-1β-induced inflammatory cell death by interacting with and inhibiting the downstream Fyn-related kinase (FRK) (176, 177). Another study has indicated that the C3-mediated protective effect on pancreatic islet β cells involves AKT activation and c-Jun N-terminal kinase (JNK) inhibition upon treatment with pro-inflammatory cytokines, such as IL-1β and IFN-γ (178). Thus, in different cell types, such as T cells, Paneth cells, and pancreatic β cells, the cytosolic C3 supports their survival and division. Additionally, C3 present in the breast milk protects suckling mouse pups from *Citrobacter rodentium*-mediated enteric infection by shaping the evolving pup gut microbiota (killing of commensal Gram-positive *Staphylococcus lentus* B3) but without affecting the production of secretory antibodies in the breast milk (179, 180). A more detailed review of C3a–C3aR interactions on different immune cells would be too cumbersome for the main text and is summarized in **Table 2**.

**Table 2:** C3aR expression (cell surface and cytosolic) on different immune cells and the impact of C3 fragments on their immune functions.

Immune cells	Extracellular C3aR	Immunological impact	Intracellular/cytosolic C3aR	Immunological impact
*Monocytes and macrophages* (181, 182)	Yes, human monocytes express C3aR but not murine monocytes (183). Human and murine macrophages express C3aR, but murine alveolar macrophages do not (184).	C3a–C3aR interaction in monocytes and macrophages may exert pro- and anti-inflammatory action (23, 185). During human macrophage differentiation, C3a induces defective M2 macrophage production as indicated by the lower expression of different M2-associated genes, such as CD206, CCL22, IL1Rα, and PPARγ, but increased expression of TNF-α and IL-6 (186). Furthermore, C3a does not alter human M1 macrophage polarization but decreases TLR4 expression and thus the LPS-induced inflammatory response (186). However, in murine peritoneal macrophages, C3a–C3aR interaction induces anti-inflammatory M2 macrophage polarization (187).	Murine brain and lamina propria macrophages express intracellular C3aR (188).	Unknown.
*Neutrophils* (181, 182)	Human neutrophils express C3aR, but the expression of C3aR on normal murine neutrophils is controversial, and its expression increases during inflammatory conditions (183, 189, 190).	Immunomodulatory action in different inflammatory and infectious diseases (23, 191–193). C3a–C3aR-induced NETosis increases coagulation and N2 polarization to promote tumorigenesis in humans, such as small intestine cancer (189).	Human neutrophils express intracellular C3aR.	Unknown.
*DCs* (194)	Yes, human and mouse DCs express C3aR depending on their origin and tissue location (183, 195). For example, murine splenic DCs lack cell surface C3aR (183, 188). Human immature pDCs express C3aR, but murine pDCs in different target organs lack C3aR, except pDCs isolated from lamina propria (LP) of the small intestine (183, 188).	C3a–C3aR interaction in DCs regulates T-cell response to alloantigens by regulating major histocompatibility complex (MHC) and co-stimulatory molecule expression and decreasing their cytosolic cAMP levels (196, 197). C3a–C3aR interaction on DCs also promotes DC recruitment at the site of inflammation with higher levels of interferons (IFNs) and prostaglandin E2 (PGE2) (198).	Unknown.	Unknown.
*Eosinophils* (181, 182)	Yes, in humans only.	C3a–C3aR axis induces eosinophil chemotaxis, degranulation, oxidative burst, and calcium (Ca^2+^) mobilization in humans (199–202).	Mouse blood, lung, and vascular adipose tissue-derived eosinophils express intracellular C3aR only (183, 188).	Unknown.
*Basophils* (182, 203)	Yes, in normal human basophils and tumor-derived basophils, but not in murine basophils (181, 182, 204).	C3a via C3aR does not induce basophil degranulation and IL-13, IL-4, histamine, and leukotriene C_4_ (LTC_4_) release (205).	Unknown.	Unknown.
*Mast cells*	Both murine and human mast cells express C3aR depending on their location, which further increases inflammatory stimuli (183). For example, naïve human bone marrow-derived mast cells (hBMMCs) do not express C3aR, which is also true for murine BMMCs and peritoneal mast cells (183, 206).	C3a via C3aR stimulation on mast cells induces their degranulation and histamine release (205, 207–209).	Unknown.	Unknown.
*Platelets*	Both mouse and human platelets express C3aR (210, 211).	C3a–C3aR axis on platelets regulates different steps of thrombus formation, such as platelet aggregation, spreading, Ca^2+^ influx, and bleeding time (210, 212). Therefore, C3a–C3aR axis activation on platelets increases the risk of thrombus formation, myocardial infarction, and stroke.	Unknown.	Unknown.
*Natural killer (NK) cells*	Human circulating NK cells express extracellular C3aR (213). Only activated mouse NK cells express C3aR (214).	C3a via C3aR also inhibits NK cell cytotoxicity (213). C3aR activation also blocks NK cell migration to the tumor microenvironment by altering localization and confirmation of lymphocyte function-associated antigen-1 (LFA-1) (214, 215).	Unknown.	Unknown.
*T cells [CD4^+^*, *CD8^+^*, *and regulatory T cells (T_regs_)]*	C3aR expression of mouse T cells is controversial (188, 216), but human activated Th1 and CD8^+^ T cells express extracellular C3aR, but not expressed by naïve T cells (217, 218).	Exogenous C3a via cell surface C3aR is critical for T-cell proliferation and differentiation (216). The cell surface C3a–C3aR interaction diminishes their immunoregulatory function (219). The cytosolic C3a and C3b generated during T-cell receptor (TCR) activation translocate to the cell surface and bind to cell surface C3aR and CD46, driving IFN-γ secretion from human CD4^+^ T cells (170, 220).	Yes, human CD4^+^ T cells in the peripheral blood also express C3aR on their lysosomes under normal conditions, which further increases during inflammatory conditions (170).	Intracellular C3a–C3aR signaling on lysosomes is critical for sustaining homeostatic survival of human CD4^+^ T cells (170, 220).
*Astrocytes*, *microglia*, *and neurons (cortical and hippocampal neurons and Purkinje cells). However*, *C3aR expression on neurons does not alter with inflammatory brain conditions*, *such as experimental autoimmune encephalitis or EAE*, *but its expression increases on microglia and astrocytes* (221–223)	Yes.	Pro-inflammatory, immune cell infiltration, or chemotaxis.	Intracellular C3aR expression in microglia and astrocytes is an intriguing question (224).	Unknown.
*Endothelial cells*	No data available for murine endothelial cells expressing C3aR, but human microvascular endothelial cells (HMECs) and primary endothelial cells express C3aR (225–227).	Immunomodulatory action depending on the stimulus/disease and target organ, as these factors are critical due to their intensity of expression and function (226–229).	Unknown.	Unknown.
*Epithelial cells*	Extracellular C3aR has not been observed in lung parenchymal, epithelial, and smooth muscle cells (SMCs) (184).	Extracellular C3a–C3aR axis synergizes with TGF-β to activate NLRP3 inflammasome for epithelial–mesenchymal transition of renal tubular epithelial cells (RTECs) during carcinogenesis (230).	Yes, retinal pigment epithelial cell (RPEC) mitochondria express C3aR, which increases during cellular stress due to the endocytic trafficking of the extracellular membrane-bound C3aR via endosomal-to-mitochondrial cargo transfer (231).	Increased mitochondrial C3aR expression and its stimulation with cytosolic C3a in stressed epithelial cells decrease ATP production by inhibiting state III ADP-driven respiration and maximal respiratory capacity (231). This may alter their metabolism, regulating survival, proliferation, and innate immune functions.
*Hematopoietic stem cells (HSCs)*	Yes (232).	Enhances effect of stem cell-derived factor-1 (SDF-1) and improves bone marrow engraftment.	Unknown.	Unknown.
*Peripheral and tonsillar B cells* (182)	No.	Unknown.	Unknown.	Unknown.
Germinal center (GC) B cells	Yes (233, 234).	C3a–C3aR axis is critical for normal GC function by controlling affinity maturation, plasma cell and memory B cell formation through mTOR, and Myc-dependent metabolic regulation (233, 234).	Yes.	Intracellular C3a–C3aR axis activates mammalian target of rapamycin (mTOR)-dependent metabolic reprogramming in GC B cells (233, 234).

#### 2.1.2 Impact of C5a on various immune cells via direct interaction with C5aRs (C5aR1 and C5aR2 or C5L2)

The C5a generated during CS pathways due to the breakdown of C5 into C5a and C5b exerts immunomodulatory actions by interacting with C5aR1 (CD88) and C5aR2/C5L2. For example, C5a–C5aR1 interaction mediates potent leukocyte chemoattraction at the site of inflammation. It induces pro-inflammatory phenotype and functions on different immune cells during sterile and infectious inflammatory conditions, such as autoimmune diseases [rheumatoid arthritis (RA) and Crohn’s disease (CD)], allergies (asthma), ischemia–reperfusion injuries, and sepsis (235–239). However, the discovery of the second C5a receptor called C5L2 [a seven-transmembrane domain G protein-coupled receptor (GPCR)] or C5aR2 in 2000 has generated controversies in the previously established pro-inflammatory functions of C5a, as C5L2 has now been considered an anti-inflammatory C5aR (240, 241). C5L2 is also considered an active metabolic receptor, and its ligand C5a and C5a_desArg_ or acylation-stimulating protein (ASP) [the degraded/desarginated C5a fragment generated via enzymatic (carboxypeptidase) degradation-dependent endocytosis], is time-, clathrin-, and cholesterol-dependent (241, 242).

Circulating ASP levels increase in people with obesity, insulin resistance or Type 2 Diabetes Mellitus (T2DM), and metabolic syndrome, which increases monocyte chemoattractant protein-1 (MCP-1) and keratinocyte-derived chemokine (KC or IL-8) from their adipocytes through C5L2 or C5aR2 interaction without impacting IL-6 and adiponectin production (243, 244). The MCP-1 and KC production from adipocytes in response to ASP/C5L2 interaction involves phosphatidylinositol-3 kinase (PI3K) and nuclear factor-kappa B (NF-κB) activation (244). However, ASP via C5L2 interaction in macrophages does not induce MCP-1 and KC production. In contrast, the adipocyte-mediated production of these cytokines in the adipose tissue (AT) increases monocyte/macrophage chemotaxis and their inflammatory function (243). For example, C5a_desArg_-induced C5L2 activation in adipocytes induces triglyceride synthesis and glucose and fatty acid (FA) uptake, which is absent in the adipocytes of C5L2 knockout mice (243–245).

C5L2 ligand binding induces their internalization and degradation, which decreases their extracellular expression level, which is a step to decrease the generation of profound or tissue-damaging complement-mediated inflammatory immune response (241). In contrast, C5aR1 internalizes ligands at a slow rate, which are further expelled back into the extracellular environment without undergoing degradation, which further aggravates the inflammatory cascade (241). Furthermore, Thr196Asn mutations in the C5L2 gene were associated with hyperlipidemia and retinitis pigmentosa (RP) in a Chinese family (246).

In humans, the GPR77 gene on chromosome 19, q13.33-13.34, is located downstream of the C5aR1 gene encoding C5aR2 or C5L2 (247). Interestingly, C5L2, belonging to C3a, C5a, and formyl Met-Leu-Ph (fMLP) receptors related to the chemokine receptor family, also binds to C3a with a moderate affinity (247, 248). However, the C3a binding to C5L2 can be easily displaced by C4a, indicating that C3a has a lower affinity to C5L2 than C5a. Moreover, the binding of anaphylatoxins (C3a and C5a) to C5L2 only increases the immune cell degranulation potential upon cross-linking high-affinity immunoglobulin E (IgE) receptor by a pertussis toxin-sensitive mechanism. C5L2 binding affinity to C5a is similar to that of C5aR1. C5L2 binds to C5a_desArg_ with a higher affinity than C5aR1 (242, 247). The C5L2 transcripts have been widely expressed in different organs, such as the spleen, testis, brain (frontal cortex, hippocampus, and hypothalamus), heart, lung, liver, kidney, adrenal gland, thyroid gland, spinal cord, ovary, and colon, and in immune and non-immune cells, like granulocytes, immature DCs, adipocytes, and skin fibroblasts, but not in monocyte-derived macrophages (MDMs) (248–253). **Table 3** shows the impact of C5a and C5aR1/C5L2 or C5aR2 interaction on different immune cells.

**Table 3:** C5aR1 and C5L2 expression (cell surface and cytosolic) on different immune cells and the impact of C5a on their immune functions.

Immune cells	Extracellular/intracellular C5aR1	Immunological impact	Extra-/intracellular C5aR2 or C5L2	Immunological impact
*Monocytes and macrophages (both murine and human)* (182)	Yes, both mouse and human macrophages express C5aR1, but the intensity of the receptor expression is organ-specific (183).	Pro-inflammatory function.	Both mouse and human macrophages express C5L2 (183).	Immunomodulatory action.
*Neutrophils* (182, 254)	Yes, both murine and human neutrophils express extracellular C5aR1 (183, 255).	C5a–C5aR1 interaction regulates their phagocytic and pro-inflammatory functions (183, 255).	Yes, both mouse and human neutrophils express C5L2 (248, 256).	C5L2 stimulation in neutrophils controls their immunological function by regulating the expression of Fcγ receptors and CD11b, thus regulating their phagocytic and inflammatory function (256).
*DCs*	Yes, murine DCs, except intestinal and pulmonary CD103^+^ DCs and plasmacytoid DCs (pDCs) (194, 255). Only activated human DCs express C5aR1, and its expression on pDCs depends on their activation status. However, human moDCs express both C5aR1 and C5aR2 (257).	DC maturation increases co-stimulatory molecule expression and IL-12 and TNF-α production and supports the generation of pro-inflammatory Th1 immune response in mice (257). However, C5a treatment of prior activated (TLR-mediated) DCs suppresses their pro-inflammatory function in both mice and humans (257, 258)	Modest expression in murine lung monocyte-derived DCs (moDCs) (60–65%) and lung CD11b^+^CD103^−^ DCs (15–20%); low C5aR2 expression is observed in intestinal CD11b^+^ and CD103^+^ DC subsets (257). C5L2 is highly expressed in immature DCs (248).	Activation of immature DCs and leukocyte recruitment (248).
*Eosinophils* (182)	Both mouse and human eosinophils express C5aR1 (259–261).	C5a–C5aR1 interaction promotes transendothelial migration of eosinophils.	Both human and mouse eosinophils express C5L2 (183, 262).	Critical role in experimental allergic asthma (EAA) (263).
*Basophils* (182)	Human basophils express C5aR1 (264, 265).	C5a–C5aR1 interaction on basophils induces their degranulation and histamine, leukotriene C_4_ (LTC_4_), IL-4, and IL-13 release (264, 266).	Unknown.	Unknown.
*Mast cells*	Murine BMMCs and peritoneal mast cells do not significantly express C5aR1 but increase with allergen/antigen/inflammogen stimuli (206). Human mast cells express C5aR1 (267).	C5a–C5aR1 interaction promotes their inflammatory function, including mast cell degranulation and histamine release, and chemotaxis to inflammatory site (267–269).	Human mast cells express C5L2 (270).	C5L2 activation in human mast cells induces the release of pro-inflammatory cytokines and chemokines (271, 272).
*Platelets*	Both human and mouse platelets express C5aR1 (211, 273).	C5a–C5aR1 interaction on platelets activates their pro-inflammatory functions, such as their degranulation and aggregation, but also negatively regulates neovascularization by releasing CXCL4 or platelet factor 4 (PF4), an antiangiogenic factor, which also induces NETosis (273–277).	Unknown.	Unknown.
*NK cells*	Human NK cells express C5aR1 extra- and intracellularly; however, intracellular C5aR1 further increases in preterm infants (213, 278). In mice, naïve NK and NKT cells do not express active extracellular C5aR1, which is seen only during inflammatory conditions, like sepsis (279, 280).	Pro-inflammatory action.	Murine splenic and circulating NK cells express C5aR2 (254). Human circulating NK cells express C5aR1 and C5aR2 intracellularly (213).	NK cell C5aR2 activation suppresses IL-12/IL-18-induced interferon (IFN-γ) production (254).
*γδT cells*	Mouse γδT cells express C5aR1 on their surface (281, 282).	C5aR1 activation on γδT cells increases IL-17 expression and, thus, their pro-inflammatory function (281, 282).	Murine γδT cells do not express C5L2.	Not applicable.
*CD4^+^ T cells*	Murine CD4^+^ T cells express C5aR1 (216, 283). Murine naïve or activated CD4^+^ T cells do not express C5aR1 (255). Human CD4^+^ T cells express intracellular C5aR1.	The locally produced C5a via cell surface C5aR1 increases the effector T-cell (T_eff_) proliferation and prevents their apoptosis by increasing Bcl-2 and decreasing Fas expression during antigen challenge (216, 283).	Central memory T cells express C5L2 (262).	Unknown.
*CD8^+^ T cells*	Human CD8^+^ T cells express C5aR1 (284).	C5a–C5aR1 axis is critical for optimal CD8^+^ T-cell response during viral infections, such as influenza A virus infection (IAV), but suppresses antitumor CD8^+^ T cells (285, 286).	Not clear.	
*Thymus-derived (natural) CD4^+^ FoxP3^+^ regulatory T cells (T_regs_)*	Murine T_regs_ express C5aR1 (219).	T_reg_ C5aR1 activation via C5a diminishes their immune regulatory function (219).	Not clear.	
*B cells*	Murine and human germinal center (GC) B cells express C5aR1 (234).	C5a–C5aR1 axis is required for optimum GC formation, effective GC B-cell activation, antibody affinity maturation, and somatic maturation (234).	Murine and human GC B cells express C5aR2/C5L2 (234).	C5L2 serves as “decoy” C5a receptor (234).
*Astrocytes*, *microglia, and neurons* (221, 287, 288). In contrast to C3aR, the C5aR expression on neurons increases during CNS inflammation (222)	Yes.	Activation of astrocyte and microglia C5aR1 by C5a binding is pro-inflammatory and induces their proliferation and astrogliosis and microgliosis (289–291)	Astrocytes and microglia.	C5a–C5aR2/C5L2 interaction in astrocytes plays a role in astrocyte-mediated neuroinflammation (289). However, C5L2 exerts neuroprotection in traumatic brain injury (TBI) and chronic neurodegenerative diseases, such as Alzheimer’s disease (AD) (292, 293).
*Endothelial cells*	HMECs and primary endothelial cells express C5aR (225–227). Mouse dermal microvascular endothelial cells (MDMECs) and capillary endothelium of their lungs also express C5aR (294)	Immunomodulatory action depending on the stimulus/disease and target organ, as these factors are critical for their intensity of expression and function (225, 226).	C5L2 expression in endothelial cells of atherosclerotic plaques (295).	C5a–C5L2 axis exerts pro-inflammatory action (increased levels of IL-1β and TNF-α) in atherosclerotic plaques (295).
*Epithelial cells* (296)	Human colonic epithelial cells express intracellular C5aR1 (297). Malignant epithelial cells, such as colorectal cancer epithelial cells, overexpress C5aR1 (298).	The intracellular C5a generated through cathepsin-D (CTSD) binds and activates intracellular C5aR1, which, via downstream C5a/C5aR1/KCTD5/cullin3/Roc-1 complex, aggravates β-catenin stability to promote colorectal tumorigenesis (297). Human retinal epithelial cells also express C5aR1 in their mitochondria, which, upon C5a binding, increase mitochondria-endoplasmic reticulum (ER) contact frequency and mitochondrial fusion, sensitizing cells to oxidative stress, promoting mitochondrial fragmentation and cell death (299).	Epithelial HeLa cells constitutively express C5L2, and its expression decreases upon treatment with IFN-γ and TNF-α (300). Keratinocytes also express C5L2 (300).	Not clear.
*Hematopoietic stem cells (HSCs)*	No (232).		No.	

## 3 CS in human pregnancy

### 3.1 CS in human fertilization

**Figure 3:**
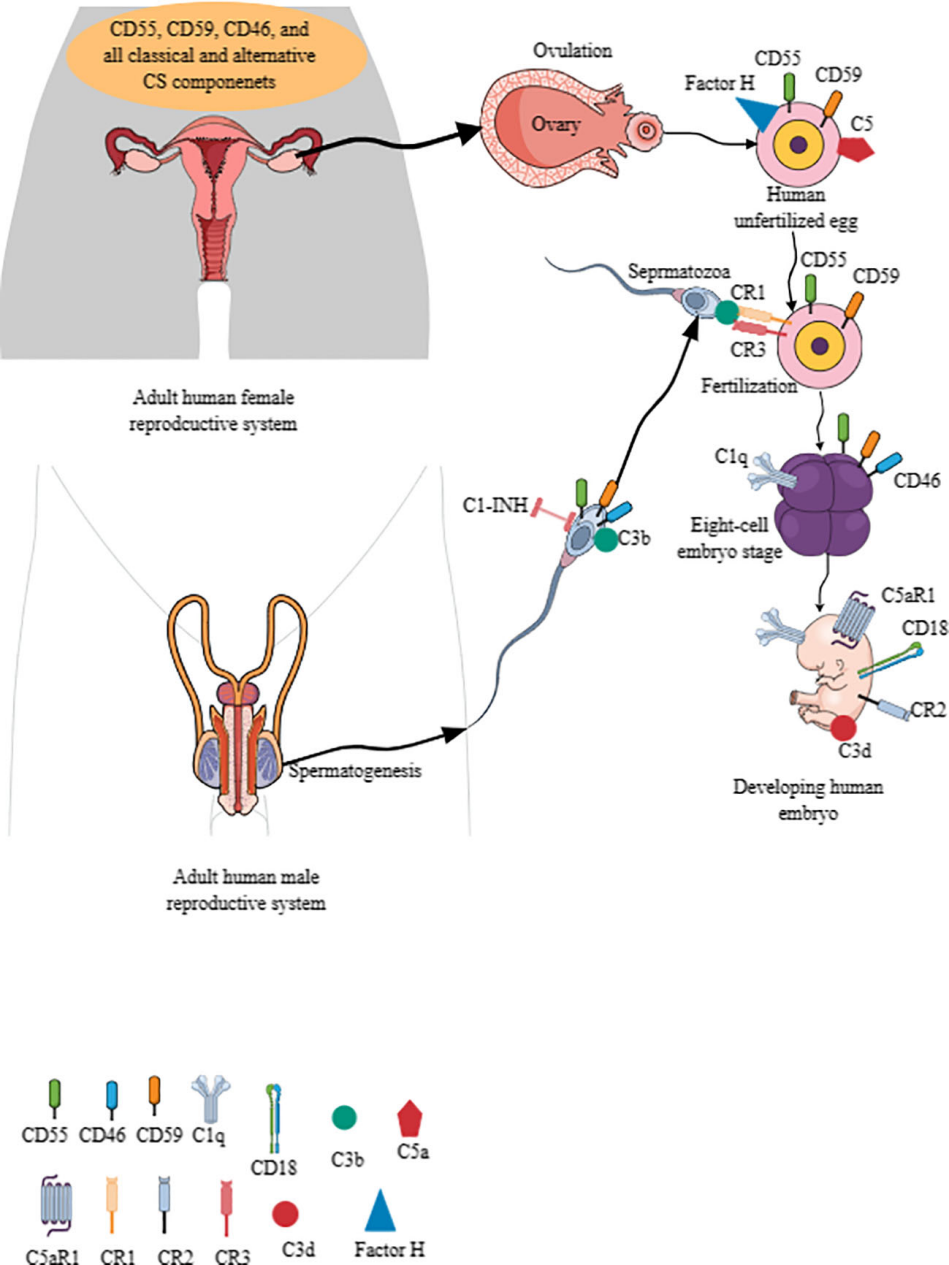
The CS signaling pathway in establishing human pregnancy. The complement components are present in human male and female reproductive tracts, and their gametes (eggs and sperms) also express different CS components. For example, CD55, C59, and C46 are present in the human uterus, ovaries, fallopian tubes, and cervix. Furthermore, the uterine, tubal, and follicular fluids have all the components of classical and alternative CS signaling pathways. Human unfertilized eggs also express CD55, CD59, factor H, C5, CR1, and CR3. Human sperms also express CD55, CD59, CD46, C3b, and C1-INH. The C3b interaction with CR1 and CR3 between sperm and ovum is critical for fertilization. Different complement components are expressed on developing embryo depending on the developmental stage. For example, CD46 appears at six-to-eight embryonic stage, which also expresses C1q. C5aR1, CD18, CR2, and C3d are also expressed by the developing embryo to check the complement activity for healthy pregnancy. Kindly see the text for details. CS, complement system.

The CS is critical for women’s reproductive health and successful pregnancy (fertilization to childbirth). For example, the human female reproductive tract (FRT), comprising the ovaries, fallopian tubes, uterine endometrium, myometrium, and cervix, expresses complement regulatory proteins (CRPs), such as CD55 and CD59 (protectin), and CD46 [membrane cofactor protein (MCP)] (**Figure 3**) (301). The CRPs (CD55, CD59, and CD46) are overexpressed in stressed endometrial cells, indicating that their endometrial cells develop a complement-mediated lysis process, modifying their inflammatory outcome in different immune-mediated inflammatory diseases (IMIDs) (302). However, all CS components/proteins of classical and alternative pathways are present in the uterine, tubal, and follicular fluids (303).

Moreover, human unfertilized eggs (plasma membrane and zona pellucida) and pre-implantation embryos express CD55/DAF and CD59, preventing them from complement attack (**Figure 3**) (304–306). Unfertilized oocytes do not express CD46, but it appears at the six-to-eight cell embryonic stage on their cell membrane as the first embryonic human gene expression begins (**Figure 3**) (304, 305). Interestingly, oocytes and pre-implantation embryos do not express complement receptor 1 (CR1 or CD35)/C3b/C4b receptor and major histocompatibility complex 1 (MHC-1) proteins (303, 304). Of note, mRNA transcripts of soluble complement inhibitors, including C4b-binding protein (C4BP beta chain), factor I, and clusterin, are present in oocytes through their eight-cell stage embryo blastomeres (306).

The mRNA transcripts of central complement activating components, such as C3 and C5, along with their activators (factor B and D, C3 activators in the alternative pathway) and other complement cascade proteins (C1s and C2) of the classical and MBL CS pathways are also present in oocytes and embryos, indicating that their transcripts remain after fertilization (306). Human embryos also express C5aR1, CR2, and CD18 (**Figure 3**). CD18/CD11b serves as CR3, and CD18/CD11c comprises CR4. C1q is present at all embryonic developmental stages (**Figure 3**) (306). The C3b/iC3b complex on the cell surface of different early embryonic developmental stages indicates targeting of the embryo by the activated complement. The inactivated C3, known as C3d, is present on the cell surface of the developing embryo, indicating that a complement activation check is crucial for embryo development (**Figure 3**) (306). However, C5 is expressed on the zona pellucida surface of the oocyte but not on the surface of blastomeres (**Figure 3**). Interestingly, oocytes and blastomeres do not have intracellular C3 and C5 but express C4bp and factor H on their cell surface (306). Factor H is also present in the zona pellucida of oocytes (**Figure 3**).

In addition to the expression of complement proteins in the human female ovum, the sperm also expresses different complement components. For example, human sperms express C1 inhibitor (C1-INH), C1qR (cC1qR and gC1qR/p33), CD46, CD55, and CD59, which may be critical for their survival and motility (**Figure 3**) (307–309). CD46, CD55, and CD59 are expressed on the inner acrosomal membrane of a human sperm, which are critical for the fertilization process but do not protect sperm from anti-sperm antibodies and complement-mediated immune attack (310). Furthermore, C1q promotes the agglutination of capacitated sperms, and C1qR (cC1qR and gC1qR) expression increases in this process (311, 312). The cleaved complement component C3b on sperm acrosome (formed during acrosome reaction) binds with CR1 and CR3 of the oocyte to facilitate the fertilization process (**Figure 3**) (313). Factor H, but not CD46, serves as a cofactor in C3b cleavage, which contributes to the fertilization process (314). Human sperm CD46 also contributes to the survival of acrosome-reacted spermatozoa in the FRT by modulating CS activation (308). The complement activation critical for the fertilization process is initiated by the C-reactive protein (CRP) and is dependent on other complement components, such as C1q, C2, and factor B (314). Hence, complement components (expressed in ovum and sperm) are critical for mammalian/human fertilization to create a new life.

### 3.2 CS in embryo implantation and spiral artery formation

The trophoblast is a local producer of complement components in the maternal decidua, serving as a primary source of complement components C3 and C4 at the maternal–fetal interface (315). Local IFN-γ at the maternal–fetal interface positively impacts trophoblast C3 and C4 production. Uterine or decidual NK (dNK) cells comprise the majority of immune cells at the time of blastocyst implantation, are the primary source of local IFN-γ during the early stages of human pregnancy, and are critical mediators of spiral artery formation (1, 316–319). Human circulating NK cells express C3aR, which migrate to the uterine microenvironment during pregnancy to serve as dNK or uNK cells, comprising 70% of the decidual lymphocyte population. The C3a–C3aR interaction on NK cells inhibits their cytotoxic action (320, 321). Additionally, C3b deposition on target cells also inhibits NK cell cytotoxicity (322). It is well known that human dNK cells exhibit lower cytotoxic activity and secrete high levels of immunoregulatory cytokines and molecules to support a healthy pregnancy (323–325). Therefore, it would be interesting to explore how homeostatic complement activation during embryo implantation influences dNK cell functions (decreasing cytotoxic function but increasing their secretory function to release immunoregulatory cytokines) to support their pregnancy functions.

Human decidual stroma widely expresses and secretes complement component C1q, which interacts with proteins expressed on decidual extracellular matrix (DEM) and promotes trophoblast adhesion and migration by activating ERK1/2 mitogen-activated protein kinases (MAPKs) to promote trophoblast invasion of decidua and placental development (326, 327). Furthermore, epithelial–mesenchymal transition (EMT) is critical for trophoblast differentiation and maternal–fetal interface establishment, as indicated by the differentiation of trophoblast [extravillous trophoblast (EVT)] cells from proximal epithelial phenotype to a distal invasive mesenchymal phenotype called interstitial trophoblast penetrating the maternal *decidua basalis* and into the maternal myometrium (328). Complement components, such as the C3a–C3aR axis, promote EMT during fibrosis and cancer metastasis (230, 329–331). Trophoblast cells also exhibit characteristics of cancer cells and pseudotumorigenesis to nourish the developing embryo (332–334). Thus, CS components are critical for embryo implantation and placental development to maintain a healthy human pregnancy.

## 4 Impact of pregnancy on maternal circulating CS components

The circulating levels of C3a, C4a, and C5a increase in normal pregnant women and remain elevated throughout pregnancy, from 20 weeks post-gestation to the newborn’s delivery (335–337). However, some studies have indicated a decrease in circulating C5a with no alteration in C3a and C4a levels in women with healthy pregnancies than non-pregnant women (338). Moreover, an early (first trimester of pregnancy) increase in circulating C3a levels is associated with adverse pregnancy outcomes (336, 339). Nevertheless, a study from China has indicated that the circulating C1q, C5a, and C5b-9 (MAC) levels in the first and second trimesters are similar to those of non-pregnant healthy women (340). In contrast, increased levels of C3, properdin, C1q, factors H and B, C4, and adipsin and decreased levels of circulating C2 and C5a have been associated with successful implantation as indicated by a study comprising Middle Eastern (Qatar) women with obesity undergoing *in vitro* fertilization (IVF)-assisted conception (341). The maternal circulating C1-INH level decreases during this period (336). Thus, in a healthy pregnancy, maternal circulating complement components, such as C3a, adipsin (FD), and C5a, increase above baseline during the second and third trimesters and remain stable afterward (340, 342). However, women with preeclampsia develop higher circulating adipsin levels later in their pregnancy (343). In addition, catalyzing the rate-limiting step of alternative CS signaling pathway activation, adipsin (FD) is also involved in MAC formation and C3a and C5a anaphylatoxin generation (27, 344, 345). Thus, increased circulating adipsin levels during pregnancy and preeclampsia indicate associated metabolic and cardiovascular changes, as circulating adipsin levels are directly associated with the metabolic and cardiovascular health of an individual (346–348).

The MBL–MASP2 activity also increases during normal pregnancy (349). Any alteration in circulating complement components beyond their regulatory/protective function at the early stages of pregnancy (first trimester) causes an abnormal pregnancy outcome (337, 350–352). Preeclampsia is one of the conditions that affect the fetus and mother, which is discussed in the following section, specifically in the context of the complement system to maintain the article’s specificity. Moreover, preeclampsia predisposes surviving women to develop hypertension, cardiovascular diseases (CVDs), and metabolic syndrome later in life (353, 354).

## 5 CS in preeclampsia

Clinically, preeclampsia is characterized by the new onset of hypertension and proteinuria in pregnant women or any other maternal signs of maternal vascular dysfunction, such as edema or dysfunction of any other organ (liver, kidney, pulmonary, cerebral, or visual) or restricted fetal growth after 20 weeks of gestation (355, 356). A comparative preeclampsia study based on American College of Obstetricians and Gynecologists (ACOG) and International Society for the Study of Hypertension in Pregnancy (ISSHP) definitions of preeclampsia at term gestational age (≥37 0/7 weeks) to identify adverse maternal and perinatal outcomes has indicated the inclusion of broad definition for preeclampsia, as it can better identify women and babies at risk of adverse outcomes (357). For example, the more inclusive ISSHP definition of maternal end-organ dysfunction seemed to be more sensitive in identifying adverse maternal and perinatal outcomes associated with preeclampsia than following the less inclusive ACOG definition (357). Moreover, the inclusion of uteroplacental dysfunction (particularly when angiogenic factors are included) to diagnose preeclampsia on its broad definition optimizes preeclampsia identification in pregnant women and babies at risk (357). In contrast, eclampsia represents severe convulsions/seizures in women with gestational hypertension or preeclampsia (355, 358). The etiology of preeclampsia, including placentation’s impact, has been described elsewhere and will not be discussed in the current article (359, 360).

It is interesting to note that preeclampsia was described in the early 20th century, while eclampsia was recognized much earlier, with descriptions dating back thousands of years (355, 361). Immunology, including the study of the innate immune system, is also a new branch of modern medicine. For example, the field of the innate immune system was revolutionized after the discovery of macrophages/phagocytosis by Elia Metchnikoff in 1882 (362). Furthermore, the functional studies associated with the CS were first described between 1888 and 1894, although evolutionarily, the CS is the most ancient component of the innate immune system, as a complement component called C3-like protein has existed a billion years ago (BYA) (12, 13, 166). Although immunological advances in preeclampsia immunopathogenesis have been made, information about the CS in preeclampsia is scarce (363–369). Furthermore, inflammation (local and systemic) also plays a critical role in preeclampsia immunopathogenesis, and the CS is a key mediator of the inflammatory process (370–373). Therefore, understanding the role of this (CS) evolutionarily ancient innate immune component in preeclampsia pathogenesis is critical to understanding its immunology.

### 5.1 Maternal circulating CS components during preeclampsia

Recent studies have indicated the alteration of CS (classical, lectin, and alternative complement activation pathways) proteins in maternal and fetal circulation and placental tissues (342, 374–376). Nevertheless, data are not equivocal for some maternal circulating complement proteins, which may be due to race, ethnicity, comorbidity, and geographical locations, as race and ethnicity also play a crucial role in the origin, pathophysiology, and outcomes of preeclampsia (377). For example, women with preeclampsia have significantly lower circulating properdin and C4 levels but higher factor B (Ba and Bb) than women with normal pregnancy, which starts to increase during early pregnancy (first trimester) (350, 374, 375, 378, 379). The decreased circulating C1q and factor H levels in patients with early- and late-onset severe preeclampsia have also been observed (379). However, another study has indicated a significant increase in circulating C1q and C4d levels in late-onset severe preeclampsia (LOSPE). C3a, C5a, and MAC levels also increase in maternal circulation with early-onset severe preeclampsia (EOSPE) and LOSPE (380–382). The placentae of pregnant women with preeclampsia express lower C3aR and C5aR levels than those of women with normal pregnancy (381, 383). Nevertheless, a positive correlation between higher serum C3a and C5a levels in women with preeclampsia with circulating angiotensin II type 1 (AT1) receptor agonistic autoantibody (AT1-AA) has been observed (381). AT1-AA is one of several mediators of hypertension during pregnancy, along with increasing soluble fms-like tyrosine kinase-1 (sFlt1) and soluble endoglin (CD105) (sEng), and endothelin-1, which are elevated in women with preeclampsia (384–386). Increased sEng and sFlt1 in EOSPE directly correlate with MAC levels and, inversely, with circulating C3a levels (382). Thus, increased AT1-AA in circulation during pregnancy may activate the CS signaling pathway to generate potent anaphylatoxins, such as C3a and C5a, to induce EOSPE. Increased circulating sFlt1 during pregnancy induces hypertension, proteinuria, and glomerular endotheliosis, which are associated with preeclampsia (387). In addition to the circulating complement component alteration during preeclampsia, the cell surface component, such as CD93 (C1qRp or C1qR1 expressed on endothelial cells), level decreases in the circulation, whereas its level increases in the serum during the first trimester of normal pregnancy (388). Therefore, further studies are needed in this direction.

A study of women with singleton pregnancies in Colombia has indicated the association between decreased maternal circulating factor H in the first trimester and spontaneous preterm birth (389). Recently, a genome-wide association study (GWAS) in Finland has identified five rare heterozygous factor H variants (L3V, R127H, R166Q, C1077S, and N1176K) only in women with severe preeclampsia (390, 391). Of these five factor H variants in women with severe preeclampsia, variants R127H and C1077S are associated with normal factor H synthesis without its release in the circulation. In contrast, variants R166Q and N1176K are associated with normal factor H secretion with reduced binding to C3b, causing dysregulated CS activation associated with severe preeclampsia (390). However, the authors could not find any defect in patients with severe preeclampsia exhibiting the L3V factor H variant. Thus, CS-associated genetic mutations can also determine women’s susceptibility or resistance to developing preeclampsia during pregnancy. The decreased maternal circulating factor H levels have also been associated with preeclampsia in women from European countries, such as the Netherlands, Finland, Norway, Italy, and the United Kingdom, without any increase in circulating anti-factor H autoantibodies (392, 393). Furthermore, the placentae [decidual stromal cells (DSCs), decidual endothelial cells (DECs), and EVTs] of women with preeclampsia express lower factor H levels (mRNA and protein) than those of women with normal pregnancy (393). In addition to factor H, other C3b regulators, such as MCP and Complement Factor I (CFI) genetic mutants [typically associated with atypical hemolytic uremic syndrome (aHUS)], in pregnant women with systemic lupus erythematosus (SLE) or antiphospholipid antibodies (APL Ab) have been identified, indicating their higher susceptibility to develop preeclampsia than normal women (394). Moreover, normal pregnant women (without SLE and APL Ab) with hypomorphic MCP and CFI genetic variants are more susceptible to developing preeclampsia than those without these variants.

Nevertheless, despite inconsistencies in different circulating CS components, a systematic meta-analysis of selected 41 studies out of a total of 456 studies has retrieved results consistently reporting reduction of C4, C3, and factor H and increase of C4d, Bb, factor D, C3a, C5a, and MAC or C5b-9 in maternal circulation during preeclampsia than in women with normal pregnancies (395). In addition to altered circulating CS components/proteins, the CR variants, such as CR3 (CD11b/18, Mac-1, or integrin αMβ2) variant M441K, display a trend toward an increased adhesion to iC3b, which is most significantly associated with preeclampsia in the Finnish Genetics of Pre-eclampsia Consortium (FINNPEC) cohort (396). The CR4 variant A251T increases C4 adhesion to iC3b, and the W48R CR4 variant decreases CR4 binding to iC3b, which may have functional consequences on the CS signaling pathway to impact preeclampsia susceptibility/resistance and severity in the population (396, 397). Even 14 variants within nine genes coding for components of the MAC or C5b-9 have a strong association with preeclampsia (398). For example, two missense variants (rs200674959 and rs147430470) of C5 are strongly associated with preeclampsia predisposition among pregnant women. Moreover, the C6 variant rs41271067 predisposes women to preeclampsia, whereas its rs114609505 variant protects against preeclampsia (398).

In addition to the classical and alternative CS components, the circulating MBL pathway components are critical for pregnancy and preeclampsia (399); for example, H-ficolin and MASP-3 of the MBL pathway of the CS decrease in women with preeclampsia (399). Ficolin-2 and ficolin-3 are also lower in pregnant women with preeclampsia than in those with normal pregnancy (400). The decreased plasma ficolin-2 level of women with preeclampsia positively correlates with circulating placental growth factor (PIGF) and inversely correlates with circulating sFlt1. However, pregnant women with preeclampsia have higher plasma MBL concentration than women with normal pregnancy (376). Women with MBL codon 54 gene polymorphism are protected from preeclampsia development (401). The protective effect of the MBL codon 54 gene against preeclampsia may be due to low MBL production, as low-MBL production genotypes are considered disease (preeclampsia) modifiers (402). Therefore, further studies are needed to establish preeclampsia’s genetic and immunological mechanisms with MBL pathway dysregulation.

Additionally, second-trimester amniotic fluids of pregnant (singleton) women have shown upregulated C3a and factor Bb before the onset of preeclampsia, indicating that CS activation during early pregnancy is associated with early-onset preeclampsia (403). Elevated C5a levels in the amniotic fluid of pregnant women developing preeclampsia have also been observed (404). Women with preeclampsia exhibit urinary excretion of the MAC because of an antiangiogenic state (high circulating sFlt1 and low PIGF and VEGF levels) associated with severe preeclampsia (405–407). Hence, altered maternal complement components during pregnancy are critical for the onset and severity of preeclampsia.

### 5.2 Placental CS components and preeclampsia

The complement proteins expressed on placental tissues are also critical for a healthy pregnancy, and their alteration plays a critical role in inducing preeclampsia (408). For example, a term placenta obtained after a healthy delivery expresses complement inhibitor C4b-binding protein (C4bBP) on its outer layer (syncytial knots of syncytiotrophoblast) that facilitates material exchange between the mother and the developing fetus (409). C4d is rarely present in the placentae of normal pregnant women, but its expression increases in syncytiotrophoblasts of women with preeclampsia (408, 410). In contrast, factor H is abundant in the placental tissue stroma of normal pregnancy, which is decreased in the placentae obtained from women with preeclampsia (409). EVTs express CD46, CD55, and CD59 in all three trimesters of normal pregnancy (411, 412). The placentae obtained from women with preeclampsia overexpress CD55 and CD59 (408). The C1q, MBL, and properdin expression in the placenta do not change between a healthy pregnancy and preeclampsia (408). However, despite no difference between control and preeclampsia in control and EOSPE patients, C1q expression decreases in LOSPE patients, which needs further investigation (408, 409). Moreover, the placental macrophages of women with preeclampsia overexpress C5a, and their trophoblasts overexpress C5aR (413). The C5a–C5aR interaction on trophoblast cells polarizes them toward an anti-angiogenic phenotype by balancing their angiogenic factors, such as sFlt1 or soluble vascular endothelial growth factor receptor-1 (sVEGFR-1) and PIGF (410, 413). Another study has indicated an increase in placental sFlt1 and PIGF in women with preeclampsia, which increases maternal circulating sFlt1 and falls post-delivery (387). The upregulated C4d, sFlt1, and MAC in the placentae of women with preeclampsia correlate well, indicating the aberrant CS activation. C5a–C5aR axis inhibition has prevented aberrant placental development by decreasing sFlt1 levels and rescued pregnancies (413, 414). Furthermore, C3a also induces the upregulation of cellular sFlt1 in human syncytiotrophoblast cells, and upregulated MAC induces its release (415). Increased sFlt1 and decreased PIGF-mediated angiogenic imbalance suppress the expression and secretion of factor H from placental endothelial cells, further activating the CS to cause endothelial cell damage and systemic vascular dysfunction, hypertension, and proteinuria during preeclampsia (416). Fetal cord blood factor B levels do not vary during healthy pregnancy and preeclampsia, and other complement components (C1q, C3, C4, and C3d) are much lower than those in healthy maternal circulation (375, 417). Nevertheless, C3d levels increase in fetal cord blood with the degree of placental inflammation, indicating their increase during preeclampsia (418).

It is interesting to mention that the placentae of women with SLE and APL syndrome show higher classical CS pathway activation, including higher C4d (a most important classical CS pathway activation marker) expression at the feto-maternal interface, leading to fetal loss and preeclampsia development, than normal healthy pregnant women (408, 419, 420). However, a very recent case–control study from Finland comprising pregnancies from 2000 to 2018 has indicated no statistical difference between pregnant women with SLE and normal pregnant women in the occurrence of preeclampsia or any other congenital malformation despite a significantly shorter duration of pregnancies and a more urgent need for cesarean section among pregnant women with SLE (421). A retrospective study comprising all SLE pregnancies during a period of 10 years (2006–2015) from a single center in Malaysia has indicated the development of complicated pregnancies, including preeclampsia, fetal losses, and the need for cesarean deliveries (422). Another retrospective study by a different group in Malaysia comprising pregnant SLE women for the period January 2008 to 2020 indicated the development of complicated pregnancies, such as SLE flares, preeclampsia, and eclampsia (423). Another retrospective study from Beijing, China, comprising 105 SLE-associated pregnancies for the period from January 1990 to December 2008, has also indicated complicated pregnancies, fetal loss, and preeclampsia development in active SLE pregnant women (424). A retrospective cohort study of 149 pregnancies in 98 women with SLE conducted over 10 years in Oman has also indicated the development of preeclampsia and eclampsia in these women along with an increase in SLE-associated pathologies, such as lupus nephritis and flares (425). The data from four retrospective studies performed in Sub-Saharan African pregnant women with SLE (137 pregnancies in 102 women) over a 26-year period have indicated a higher incidence of preeclampsia and aggravation of SLE symptoms, such as lupus nephritis and SLE flares, which further increased adverse pregnancy outcomes, including preeclampsia (426). This difference [geographical and ethnic origin (Europe, Asia, and Africa)] indicates the genetic and environmental impact on SLE and other autoimmune conditions affecting pregnancy outcomes, including preeclampsia, which must be explored. SLE and APL syndrome-mediated immune alteration, including CS pathway association with pregnancy loss and preeclampsia discussion, is beyond the scope of the current article and has been discussed elsewhere (427–429).

### 5.3 Mechanisms of CS pathway activation during preeclampsia

We have discussed earlier that altered CS signaling pathways are critical players in the onset and severity of preeclampsia. However, we do not know the triggers activating the CS pathway to induce inflammatory consequences that support and aggravate preeclampsia pathogenesis and outcome. For example, maternal hypertension and proteinuria (endothelial dysfunction) after 20 weeks of gestation are significant characteristics of preeclampsia, along with increased platelet aggregation and the hyperactivation of the coagulation system (430, 431). The pathogenesis of preeclampsia varies in nulliparous women compared with women with pre-existing vascular diseases, metabolic syndrome, multifetal gestation, and/or previous preeclampsia. However, some pregnant women with HELLP (hemolysis, elevated liver enzymes, or low platelet counts) syndrome (10%–15%) or eclampsia (38%) may not exhibit hypertension or proteinuria, which are associated with higher rates of maternal and fetal morbidities than in mild preeclampsia (432, 433). Interestingly, HELLP syndrome exhibits elevated maternal CS pathway activation as indicated by the increased systemic levels of different complement components, such as C5a, MAC, CFH, and CFH-related 1 and 3 (CFHR1 and 3), which are comparable to preeclampsia systemic values of complement components (434–437).

Furthermore, HELLP syndrome patients with complement gene variants exhibit poorer clinical outcomes than those with no complement gene variants. These patients have higher complement mutation frequency, including rare germline mutations in the alternative CS pathway (CFHR1, CFHR3, CFI, CFB, and CD46) than women with preeclampsia, having similar rates of variants as the control population (438, 439). Thus, pregnant women with complement gene variants are more likely to progress from preeclampsia to HELLP syndrome, where gene variants and pregnancy provide first and second hits, like other complement disorders, such as aHUS and paroxysmal nocturnal hemoglobinuria (PNH). Furthermore, a clinical trial with eculizumab, a long-acting human monoclonal antibody targeting C5 to block its cleavage to C5a and C5b, has shown positive results in phase 1 clinical trials of pregnant women with early-onset HELLP syndrome (440). Hence, CS activation is critical for the pathogenesis and severity of preeclampsia and HELLP syndrome. Therefore, it is critical to identify factors that dysregulate the CS activation during preeclampsia and its severe forms.

#### 5.3.1 Maternal factors associated with increased risk of preeclampsia and their association with complement dysregulation

Women facing infertility associated with polycystic ovary syndrome (PCOS) and recurrent pregnancy loss (RPL; defined as ≥3 consecutive embryonic losses before 10 weeks’ gestation or ≥2 fetal losses after 10 weeks’ gestation) are more prone to develop preeclampsia (441). The immune system plays a critical role in the pathogenesis of PCOS and RPL, and the CS is a critical component of the immune system. Studies have shown that high maternal circulating C3 and C4 levels via CS signaling pathway activation before conception are associated with RPL independent of MBL CS pathways and other components of immunity, such as B cells and antibodies (442–445). Furthermore, women with specific mutations in C4BP carrying C4BP gene polymorphism R120H also suffer from RPL due to decreased C3b degradation that elevates their circulating C3b level (446). Another study has indicated several C3 gene variants associated with defective secretion/function of C3 in European women (*n* = 192) who suffered from RPL (447). Thus, dysregulated CS activation, such as the alternative CS signaling pathway in women who suffered RPL previously, predisposes them to develop preeclampsia during a successful pregnancy due to their altered CS signaling pathway, even in the presence of healthy placental development. Furthermore, fasting circulating complement components, such as C3 and C3a (desArg), are higher in PCOS women with insulin resistance, which increases to a similar extent in the control and PCOS groups (448). However, higher factor H levels are present in the circulation in PCOS women with obesity (448, 449).

Non-obese, non-insulin-resistant women with PCOS have higher systemic alternative and classical CS signaling pathway components, such as C3, iC3b, properdin, and C4 levels (450). Further study has indicated that upregulated alternative CS pathway components, such as C3, properdin, factor B, and factor I, are elevated in non-obese patients with PCOS, which further increases with obesity (449, 450). Systemic C5a levels are also increased in normal-weight women and women with obesity suffering from PCOS (449). Hence, activation and terminal CS pathway components are altered in PCOS women, which increases their propensity to develop RPL. Thus, we can speculate that infertile women (due to RPL and PCOS) with otherwise normal sexual function and immune components may have altered CS components, which increases their propensity to develop preeclampsia.

Elevated body mass index (BMI) and obesity are other definite preeclampsia risk factors (431, 451–454). AT comprises adipocytes and stromal vascular fraction (SVF), containing different cell types, such as preadipocytes, fibroblasts, and immune cells (macrophages and T cells). Adipocytes are the primary source of FD, a critical player in the alternative CS signaling pathway activation (455). Along with FD, other alternative CS components, such as C3, factor B, factor H, factor I, and properdin, are overexpressed in ATs, which increase with BMI and obesity status (455). Furthermore, adipocytes express C3aR and C5aRs (C5aR1 and C5aR2), which, via their corresponding ligands (C3a and C5a), increase the local and systemic inflammation along with increasing insulin resistance (244, 456, 457). Women with obesity with elevated circulating Bb (active protease, generated during the alternative CS signaling pathway) and C3a levels compared with the control group are more likely to develop preeclampsia (458, 459). Thus, women with obesity, high BMI, and insulin resistance or glucose tolerance have hyperactivated alternative CS signaling, which predisposes them to develop preeclampsia during their pregnancy. Hence, pre-pregnancy CS component dysregulation due to the above-mentioned factors in women increases their chances of facing preeclampsia during their pregnancy.

## 6 Future perspective and conclusion

Preeclampsia is a disease that specifically occurs during pregnancy; therefore, the placenta and dysregulated maternal immune response are key factors for its pathogenesis. However, immunological advances in pregnancy and preeclampsia have now clarified that poor placentation is not the only driving force behind preeclampsia pathogenesis but rather serves as a critical factor for preeclampsia predisposition (431). The degree of maternal physiological reaction, including the immune response severity, determines the predisposition to preeclampsia and its severity. For example, the immune system governs the effective allotransplantation of the fetus in the uterus of the pregnant woman by modifying the maternal local (uterine) and systemic immune response, vascular, and coagulatory functions, which are further governed by the hormonal and psychogenic changes taking place in a pregnant woman (460–465). Hence, any pre-pregnancy immune dysfunction can be lethal to the future mother and developing fetus, as the CS is the first and rapid immune component of innate immunity; therefore, its homeostatic levels during pregnancy are critical for a healthy outcome.

Hypertension development during pregnancy is a critical factor in the development of preeclampsia. A study has indicated systemic elevation of the clusterin (a complement regulatory protein) in pregnant women during pre-hypertension disorder of pregnancy (pre-HDP) development, which proved to be the critical factor for HDP development (466). Further studies have indicated increased clusterin systemic levels before the onset of preeclampsia clinical symptoms in pregnant women that increase with preeclampsia severity (467–469). Moreover, clusterin plays a critical role in the decidualization process by interacting with the triggering receptor expressed on myeloid cells 2 (TREM-2) receptor expressed on decidual cells, and its alteration may impact placental development, including trophoblast invasion (467, 470). The higher clusterin levels in the placenta inhibit MAC formation; therefore, it will be interesting to investigate the systemic clusterin and MAC levels during normal human pregnancy and preeclampsia.

Furthermore, aberrant and persistent CS activation (local and systemic) elevates systemic MAC or C5b-9 and C1q, causing systemic vasculitis or thromboinflammation that impairs endothelial function, which may cause hypertension (471–476). Thus, aberrant CS activation in pregnant women and their placentae may induce endothelial damage that may cause hypertension and elevate circulating endothelial cells, further aggravating the CS, predisposing them to preeclampsia, and increasing its severity (476, 477). For example, fetal endothelial cell damage and CS dysregulation (elevated MAC and C3a levels but decreased factor H and Bb) have been observed in pregnancies complicated by preeclampsia (478).

C3 is a critical component in hypertension pathogenesis due to its maintenance effect on undifferentiated mesenchymal stem cells (MSCs), and maternal and placental C3a levels are upregulated in women with preeclampsia, indicating aberrant CS activation (479). Furthermore, increased maternal circulating C5a in women with preeclampsia is positively correlated with maternal blood pressure and arterial stiffness (413). Targeting the CS during preeclampsia may prevent associated organ damage, such as renal manifestations, as the kidneys are among the most affected organs in preeclampsia (480). Hence, CS proteins must be checked for a healthy pregnancy.

Women with preeclampsia also exhibit a hypercoagulable state than women with normal pregnancy at an early stage of the disease (481). Women with preeclampsia have elevated circulating levels of factor VIII, von Willebrand factor (vWF; due to endothelial cell damage/inflammation), the thrombin–antithrombin complex (TAT), D dimers, soluble fibrin, and thrombomodulin levels than women with normal pregnancy (481–483). Increased fibrin deposition in women with preeclampsia occurs in the glomerulus sub-endothelium, spiral arteries, decidual components, and occlusive lesions of placental vasculature (484, 485). Fibrin deposition activates the classical CS signaling pathway by interacting with C1q by covalent interaction mediated by FXIIIa (53). However, this fibrin–C1q interaction is antagonized by factor H, which is downregulated in women with preeclampsia.

The activated CS signaling pathway in patients with preeclampsia can stimulate the extrinsic coagulation system pathway to form thrombin by increasing the tissue factor (TF) activity on different cells, such as endothelial cells, as evolutionarily the CS and coagulation system have a common origin and interact to maintain homeostasis and hemostasis (486–489). Moreover, overproduced plasmin, a protease generated in response to thrombin production and fibrin deposition, also serves as C5 convertase and cleaves C5 into C5a and C5b to induce the inflammatory cascade and the assembly of procoagulant C5b-9 or the MAC (490). In addition to C5 cleavage, plasmin also cleaves C3 into C3a, which is upregulated in women with preeclampsia (491). Furthermore, inflammatory events, including organ injuries, complement (increase in C5a levels), coagulation (thrombin–antithrombin complexes), activation, and cross-talk, are very early events, which have also been reported in patients with preeclampsia (491). Several other coagulation pathway components, such as Factor Xa, thrombin, FIXa, and FXIa, also cleave C3 and C5 into biologically active C3a and C5a capable of exerting their pro-inflammatory effects (491, 492). Further maternal proteomics-based study has indicated the increased deposition of C5b-9 or the MAC and vWF in the endothelial cells of women with early-onset severe preeclampsia, indicating that the complement and coagulation systems are the critical pathways for early-onset severe preeclampsia (493). Thus, aberrant complement activation not only dysregulates immune homeostasis but also affects the coagulation system, hypertension, and metabolism to initiate and increase the severity of preeclampsia.

Moreover, increased circulating C3a levels have been observed in women with depression; therefore, it may be interesting to investigate whether increased circulating C3a levels predispose women to develop preeclampsia upon getting pregnant (494). Increased circulating C3a levels during pregnancy are a critical biomarker not only for preeclampsia prediction but also for depression during pregnancy, as the Edinburgh postnatal depression scale (EPDS) alone is not perfectly sufficient to detect major depressive disorder during pregnancy (495). Interestingly, more than 10% of pregnant women in high-income countries, such as the United States, have depression during pregnancy (496).

### 6.1 Conclusion

Measuring different circulating complement proteins in pregnant women may serve as a biomarker for early preeclampsia detection. Targeting the CS in pregnant women with preeclampsia will complement normal pregnancy and associated organ damage. Understanding CS signaling during preeclampsia will further help to track future maternal health issues, such as metabolic, cardiovascular, and neurologic disorders in survivors. Complementing helps in a healthy pregnancy, but decomplementing will equip us to fight against preeclampsia and other future health issues in preeclampsia survivors. Therefore, future studies are warranted to understand the CS signaling pathways’ alteration and their mechanism of action in human pregnancy and preeclampsia.
